# Activated Bone Marrow-Derived Macrophages Eradicate Alzheimer's-Related Aβ_42_ Oligomers and Protect Synapses

**DOI:** 10.3389/fimmu.2020.00049

**Published:** 2020-01-31

**Authors:** Songlin Li, Eric Y. Hayden, Veronica J. Garcia, Dieu-Trang Fuchs, Julia Sheyn, David A. Daley, Altan Rentsendorj, Tania Torbati, Keith L. Black, Ueli Rutishauser, David B. Teplow, Yosef Koronyo, Maya Koronyo-Hamaoui

**Affiliations:** ^1^Institute of Neuroscience and Chemistry, Wenzhou University, Wenzhou, China; ^2^Institute of Life Sciences, Wenzhou University, Wenzhou, China; ^3^Department of Neurosurgery, Cedars-Sinai Medical Center, Maxine-Dunitz Neurosurgical Institute, Los Angeles, CA, United States; ^4^Department of Neurology, David Geffen School of Medicine at UCLA, Mary S. Easton Center for Alzheimer's Disease Research at UCLA, Brain Research Institute, Molecular Biology Institute, University of California, Los Angeles, Los Angeles, CA, United States; ^5^Board of Governors Regenerative Medicine Institute, Cedars-Sinai Medical Center, Los Angeles, CA, United States; ^6^Department of Biomedical Sciences, Cedars-Sinai Medical Center, Los Angeles, CA, United States; ^7^College of Osteopathic Medicine of the Pacific, Western University of Health Sciences, Pomona, CA, United States; ^8^Department of Neurology, Cedars-Sinai Medical Center, Los Angeles, CA, United States

**Keywords:** Alzheimer's disease, neurodegeneration, immunomodulation therapy, amyloid-beta, regeneration, synaptogenesis

## Abstract

Impaired synaptic integrity and function due to accumulation of amyloid β-protein (Aβ_42_) oligomers is thought to be a major contributor to cognitive decline in Alzheimer's disease (AD). However, the exact role of Aβ_42_ oligomers in synaptotoxicity and the ability of peripheral innate immune cells to rescue synapses remain poorly understood due to the metastable nature of oligomers. Here, we utilized photo-induced cross-linking to stabilize pure oligomers and study their effects vs. fibrils on synapses and protection by Aβ-phagocytic macrophages. We found that cortical neurons were more susceptible to Aβ_42_ oligomers than fibrils, triggering additional neuritic arborization retraction, functional alterations (hyperactivity and spike waveform), and loss of VGluT1- and PSD95-excitatory synapses. Co-culturing neurons with bone marrow-derived macrophages protected synapses against Aβ_42_ fibrils; moreover, immune activation with glatiramer acetate (GA) conferred further protection against oligomers. Mechanisms involved increased Aβ_42_ removal by macrophages, amplified by GA stimulation: fibrils were largely cleared through intracellular CD36/EEA1^+^-early endosomal proteolysis, while oligomers were primarily removed via extracellular/MMP-9 enzymatic degradation. *In vivo* studies in GA-immunized or CD115^+^-monocyte-grafted APP_SWE_/PS1_ΔE9_-transgenic mice followed by pre- and postsynaptic analyses of entorhinal cortex and hippocampal substructures corroborated our *in vitro* findings of macrophage-mediated synaptic preservation. Together, our data demonstrate that activated macrophages effectively clear Aβ_42_ oligomers and rescue VGluT1/PSD95 synapses, providing rationale for harnessing macrophages to treat AD.

## Introduction

Alzheimer's disease (AD) is a progressive, incurable and fatal neurodegenerative disorder and the most frequent cause of senile dementia (1, 2). AD is characterized by severe synaptic and neuronal loss resulting in gradual behavioral and cognitive decline (3, 4). Early pathological changes in AD involve accumulation of amyloid β-protein (Aβ) in the brain and retina (5–11), with the amyloidogenic 42-residue long alloform (Aβ_42_) viewed as the most pathognomonic (5, 12). These Aβ_42_ peptides have been shown to rapidly aggregate into insoluble fibrils that form the plaques in AD brains, but also to assemble into non-fibrillar soluble oligomers, believed to be highly neurotoxic (8, 13–20). While there is a weak correlation between cerebral plaque density and severity of AD, Aβ_42_ oligomers appear to closely associate with synaptopathy and cognitive impairment (21, 22). Specifically, growing evidence demonstrates the detrimental effects of Aβ_42_ oligomers on synaptic plasticity, spine morphology and density (13, 23), axonal transport (24–26), and cognition (8, 15, 17, 22, 27–29). These findings incentivize strategies that eradicate Aβ_42_ oligomers in order to preserve synapses and cognitive function (30–34).

Multiple mechanisms mediate cerebral Aβ clearance, including phagocytosis and enzymatic degradation by innate immune cells such as microglia and bone marrow (BM)-derived monocytes (Mo) and macrophages (MΦ) (35, 36). However, under a chronic neuroinflammatory condition typical for AD, microglia exhibit a compromised phenotype, with diminished ability to clear Aβ (37, 38), further contributing to damaging inflammatory processes, and excessively pruning synapses (39–46). Nevertheless, unlike brain-resident microglia, BM-derived Mo/MΦ appear to have an increased capacity to remove Aβ fibrils, as shown by studies in both cell cultures and animal models (47–64). Indeed, various pre-clinical studies from our group and others have supported an emerging paradigm shift for the therapeutic potential of immunomodulation via enhanced cerebral recruitment of BM-derived Aβ-clearing macrophages (9, 35, 50, 52, 58, 62–70). In particular, we demonstrated that successful delivery of therapeutic Mo/MΦ to the brains of APP_SWE_/PS1_dE9_ double-transgenic (ADtg) mice can be achieved via immunization with altered myelin-derived antigens (e.g., glatiramer acetate – GA; also known as Copaxone^®^) or peripheral-blood enrichment with BM-derived CD115^+^-monocytes (Mo^BM^) (50, 52, 58, 63, 70). In the immunized mice, cerebral MΦ were found to be highly-phagocytic, pro-healing, and anti-inflammatory, profoundly alleviating cerebral Aβ_40_ and Aβ_42_ burden, reducing microgliosis and astrocytosis, and ultimately improving cognitive functions. Further, GA induced macrophage-mediated recognition and phagocytosis of fibrillar Aβ_42_ (63, 70). However, the potential of clearing the non-fibrillar, soluble Aβ_42_ oligomeric forms by macrophages requires further investigation.

Experimental and clinical studies have indicated that the cognitive decline seen in AD patients and animal models is related to early pathological processes occurring in entorhinal cortex layers 2 and 3 (71–75). These layers play a crucial role in connecting brain cortical regions to the hippocampus. Our immunomodulation approach of recruiting Mo/MΦ to brain sites of Aβ deposits substantially reduced AD-related pathology within these regions (63, 70). Importantly, selective ablation of peripheral monocytes, either by diphtheria toxin-targeted depletion or Ccr2-mediated inhibition of cerebral monocyte infiltration, exacerbated cortical and hippocampal Aβ pathology in ADtg mice (52, 53). In addition, our preliminary data suggested a presynaptic preservation concomitant with Aβ-plaque reduction in hippocampi of these murine models (63). Yet, the underinvestigate role of peripheral Mo/MΦ in preservation of synaptic and neurite integrity, especially postsynaptic terminals within AD-relevant cortical regions, calls for broader exploration due to promising clinical implications of immunomodulation intervention strategies for AD.

Here, we applied photo-induced cross-linking of unmodified proteins (PICUP) to assess the impact of intrinsically metastable and polydisperse Aβ_42_ oligomers on neurons and to test the ability of macrophages to recognize, bind to, and remove them (76, 77). This technique allowed us to produce pure, stabilized assemblies of low-n Aβ_42_ oligomers (XL-oAβ_42_). The precise detrimental impact of these oligomeric conformers, as compared with preformed fibrils (fAβ_42_), was determined on synaptic density, neuritic arborization length, and neuronal function in primary (P1) cortical neurons. Furthermore, synaptic and neurite protection against Aβ_42_ oligomers was explored in co-cultures of cortical neurons accompanied with BM-derived MΦ or GA-activated MΦ. Next, the innate immune mechanisms of eliminating pathogenic Aβ_42_ assemblies and subsequently attaining synaptic rescue were studied. Finally, in ADtg mice, we determined synaptic loss as well as preservation of pre- and postsynaptic terminals following macrophage-mediated immunomodulation interventions in predefined hippocampal and cortical subregions.

## Materials and Methods

### Mice

Alzheimer's disease double-transgenic (ADtg) B6.Cg-Tg (APPswe, PSEN1_ΔE9_) 85Dbo/J mice and their age-matched wildtype (WT) C57BL/6J littermates were purchased from Jackson Laboratories (MMRRC stock 34832-JAX) and then bred and maintained at Cedars-Sinai Medical Center. All animals in this study have a C57BL/6 congenic background. Two cohorts of 10-month-old males, WT and ADtg (n = 6 per experimental group), were used. The first cohort underwent weekly s.c. GA immunization or monthly i.v. CD115^+^-Mo^BM^ injections for a duration of 2 months. Control groups were comprised of either naïve WT mice or monthly i.v. PBS injected ADtg mice. At the completion of the experiment, mice underwent behavioral testing at 12 months of age, then were perfused, under deep anesthesia, with ice-cold 0.9% saline solution containing 0.5 mM EDTA, and brains were collected for analyses. Harvested brains were cut in half. One hemisphere was post-fixed overnight in phosphate-buffered saline (PBS) containing 2.5% paraformaldehyde. Tissue was cryoprotected and stored in PBS containing 30% sucrose and 0.1% sodium azide. Brains were cut coronally in 30 μm serial sections for histology. The second hemisphere was further snap-frozen for biochemical and molecular assays as described previously (63). The second cohort underwent perfusion as described above for tissue collection.

### Culture and Co-culture of Primary Cortical Neurons and Macrophages

Cortical neuronal cultures were prepared from postnatal day 1 C57BL/6 mice (78, 79). Neonates were decapitated, and their brains were removed. The cerebral cortex was dissected, stripped of meninges, dissociated with a combination of calcium and magnesium free Hank's balanced salt solution (HBSS; Life Technologies) containing 0.2% w/v papain suspension (Worthington Biochemical), and digested for 12 min at 37°C. The triturated cells were passed through a 70 μm strainer and counted. The cells were plated in laminin and poly-D-lysine-coated coverslips (BD Biosciences) at a density of 8 × 10^4^ cells per ml (in 24-well plates) in NbActiv4 (BrainBits), then supplemented with 100 units/ml penicillin and 100 μg/ml streptomycin. The purity of primary neurons was about 92–94% (78). Bone marrow-derived macrophages were obtained as described previously (63). Briefly, bone marrow cells were isolated from the femurs and tibiae of 8- to 16-week-old C57BL/6 mice, differentiated into macrophages by incubation in complete RPMI-1640 (Life Technologies), and supplemented with 10% fetal bovine serum (Life Technologies) and 20 ng/ml macrophage colony stimulating factor (M-CSF; PeproTech) for 5 days. Primary cultures of macrophages were then suspended in NbActiv4 and co-cultured with primary cortical neurons (ratio of 1:1) per well in 24-well tissue-culture plates for 2 days. Primary neurons and/or macrophages were incubated overnight with 100nM of scrambled (s), fibrillar (f), or oligomeric (o) Aβ_42_ (see below “Preparation of Aβ_1−42_ oligomers, fibrils and Scrambled Aβ_42_”). Additional groups included those with macrophages treated with glatiramer acetate (GA; 30 μg/ml, TEVA Neuroscience) for 24 h. Experiments were carried out at day 9 of seeding cortical neurons starting with synapse formation and maturation (78). Cultures were washed in PBS and then fixed in 4% formaldehyde for 20 min at room temperature for further experimental procedures.

### Microelectrode Array Recording

Cortical neuronal cultures (as described above) were plated in a M768-GLx 12-, 24-, or 48-well plate (Axion Biosystems). Preformed Aβ_42_ (100 nM) were added to the culture on day 9. Simultaneous recordings from 64 extracellular electrodes per well were made using the Maestro (Axion BioSystems) microelectrode array (MEA) system at a constant temperature of 37°C. Data were sampled at 12.5 kHz, digitized and analyzed using Axion Integrated Studio software (Axion BioSystems) where spike events were detected using an adaptive spike detection threshold of 5.5 SD for each electrode with 1 s binning. Detected waveforms were then further sorted usingOffline Sorter v4 software (Plexon) and exported through NeuroExplorer (Nex Technologies) into MatLab for waveform analysis using custom code. All data is presented as means ± s.e.m. and include recordings from 5 independent experiments.

### Amyloid-β_42_ Determination by Sandwich ELISA

At day 7, macrophages were incubated for 30 min or 24 h with fibrillar (f) or oligomeric (o) Aβ_42_ and thereafter culture medium was collected. Total protein levels in the supernatant were determined using the Pierce BCA Protein Assay Kit (#23227; Thermo Scientific). The Aβ_42_ levels in the supernatant were assessed using a sandwich ELISA for an anti-human Aβ_42_ end-specific kit (KHB3442, Invitrogen; which does not recognize mouse Aβ nor human Aβ_40_/Aβ_43_). The kit was used according to the manufacturer's instructions. This assay is based on a combination of two antibodies specific for the N- and the COOH-termini of Aβ_42_ sequences. The bound rabbit anti-COOH-terminus was detected with a horseradish peroxidase-labeled anti-rabbit antibody and was read at 450 nm using a microplate reader (Spectra Max 384 plus, Molecular Devices). Concentrations detected at 24 h incubation were normalized to basal levels at 30 min incubation.

### Immunocytohistochemistry and Quantification of Synapses and Neurites

Primary neurons/macrophages in coverslips and brain coronal cryosections at bregma −2.50, −2.65, and −2.80 mm (80) per animal were washed in PBS and then treated with a permeabilization/blocking solution containing 20% normal horse serum (Invitrogen) and 0.05% Triton X-100 (Sigma-Aldrich). Cells or tissue sections were stained overnight at 4°C with combinations of presynaptic marker VGluT1 (Chemicon, 1:6,000), postsynaptic marker PSD95 (Abcam, 1:600; for synapses), or neuron-specific class III beta-tubulin/Tuj1 (Abcam) or Tubb3 (BioLegend; for neurites; Supplementary Table 1) in 2% blocking solution in PBS. Secondary antibodies of Cy3 donkey anti-guinea pig or anti-mouse or Cy5 donkey anti-rabbit (Supplementary Table 1) were incubated for 1 h at room temperature. The samples were washed in PBS and mounted using ProLong^®^ Gold with DAPI (Life Technologies). All groups were immunostained using identical procedures. Negative controls were processed using the same protocol with the omission of the primary or secondary antibodies to assess nonspecific labeling. There was no specific staining with these negative controls. Microphotographs were shot using a Carl Zeiss Axio Imager Z1 fluorescence microscope equipped with ApoTome (AxioVision 4.6.3, Carl Zeiss). The parameters for scanning were consistent across all groups.

Synaptic and neuritic quantification of primary cultures *in vitro* was carried out from 16 images, each coverslipped at a 40 × objective lens. At least 2 coverslips, 32 images, and 150 neurons for each condition were analyzed. For synaptic analysis *in vivo* and to cover the hippocampal area, 3 of the same rectangular fields (90 × 70 μm) under 100 × oil objective lens were precisely selected in the lateral and medial blade molecular layer (ML) of the dentate gyrus (DG), the stratum lacunosum-moleculare (SLM), the stratum radium (SR) and the stratum oriens (SO) of cornu ammonis 1 (CA1) in each condition, respectively. In addition, 2 of the same fields were carefully chosen in layers 2 and 3 of the entorhinal cortex (ENT). Fifteen optical sections per field, 15 fields per hippocampal area, 4 fields per entorhinal cortex per section, and 855 total images per brain were analyzed. Single optical section images at 0.25 μm intervals and 3.75 μm Zeiss ApoTome high-resolution scans were performed. Synaptic puncta number and synaptic immunoreactive (IR) area were quantified using Puncta Analyzer (81, 82) and ImageJ (NIH) macro and batch process. Total neurite length was measured using the NeuriteTracer program (83). Briefly, the cultures were immunostained with Tuj1 for neurite and NeuN for the neuronal nucleus. For each condition, at least 150 primary neurons, 32 images in random fields from 2 coverslips in 2 independent experiments were analyzed. The NeuriteTracer was utilized to detect the neurites strongly stained for Tuj1. Following optimization of parameters to separate neurites from the neuronal cell body and tracing the neurite through skeletonization, positively labeled neurites and respective lengths were quantified (Figure 3B). The observer was blind to the treatment conditions. Average puncta number, synaptic area, and percentage of the area per image or per neuron were calculated for each condition.

### Primary Cultures of Bone Marrow-Derived Macrophages and the Phagocytosis Assay

To test amyloid-β phagocytosis by macrophages, in repeated experiments monocytes were isolated from the bone marrow of wild-type mice (*n* = 18 mice) and differentiated into macrophages by 7-day cultivation in complete RPMI-1640 medium (#21870; Life Technologies) with 10% serum and 20 ng/ml MCSF (#315-02; PeproTech). Macrophage primary cultures were then plated at 1.2 × 10^5^ cells per well (3–4 wells for each condition) in 24-well tissue-culture plates on glass coverslips overnight. Next, macrophages were either treated with 30 μg/ml GA (Copaxone^®^ TEVA Neuroscience) for the duration of 1, 3, or 24 h, or not treated (control group). Before addition of f/o/sAβ_42_ or vehicle, the cells were chilled in a 4°C ice bath for 5 min; immediately after addition of the preformed Aβ_42_ (100 nM), the plates were centrifuged at 515g in 25°C followed by incubation at 37°C for 30 or 60 min. The cells were then rinsed with Aβ-free medium to remove non-incorporated Aβ and later washed twice with PBS. Methanol (99.8%) at −20°C for 20 min or 4% paraformaldehyde at room temperature for 12 min were used for fixation of the cells followed by repeated washes with PBS. For immunostaining, the cells were first stained using the mouse anti-human amyloid-β mAb clone 6E10 (1:100; SIG-39320; Covance), rat anti-CD36 mAb clone MF3 (1:200; ab80080; Abcam), rat anti-CD204 scavenger receptor type I/II (SCARA1) mAb (1:100; MCA1322; AbD Serotec), rabbit anti-EEA1 pAb (1:100; Millipore #07-1820) and goat anti-MMP-9 pAb (1:100; AF909; R&D systems; Supplementary Table 1). Secondary polyclonal antibodies included donkey anti-mouse, anti-rat, anti-rabbit, and anti-goat conjugated with Cy2, Cy3, or Cy5 (1:200; Jackson ImmunoResearch Laboratories). The cells were mounted using ProLong^®^ Gold with DAPI (Molecular Probes, Life Technologies; Supplementary Table 1). Several fields (minimum n = 5 randomly selected per group) were obtained from each well using a Carl Zeiss Axio Imager Z1 ApoTome-equipped microscope (an average of 120 cells in each field). Images were obtained using the same exposure time on each occasion. The fluorescent signal and its total area were determined and quantified by the conversion of individual images to greyscale and standardizing to baseline using histogram-based thresholds with NIH ImageJ software. The “mean area per cell” was a result of a numerical average of the individual cell's immunoreactive area per field. The “area/cell” measures the total fluorescent signal (area) divided by the total number of cells (DAPI count) of the same field (image). For all experiments, the investigators were blinded to the treatment condition. Colocalization of EEA1-6E10 (puncta analysis) was performed as synaptic puncta number analysis described above (81, 82).

### Preparation of Aβ_1−42_ Oligomers, Fibrils, and Scrambled Aβ_42_

Filtration was used to prepare LMW Aβ_42_ as described (84). Microcon YM-30 filters (EMD Millipore) were washed in 200 μl of distilled deionized water. Aβ was dissolved in 10% (v/v) 60 mM NaOH and 90% (v/v) 10 mM phosphate buffer, pH 7.4, at a concentration of 1 mg/ml. The solution was sonicated for 1 min and then placed into the washed filter. Following centrifugation at 14,000 × g for 20 min, the filtrate, LMW Aβ_42_, was collected. This LMW Aβ was stabilized using photo-induced cross-linking of unmodified proteins (PICUP) (76, 85–87) to yield a distribution of “oligomeric Aβ.” Briefly, 2 mM Tris(2,2′-bipyridyl)di- chlororuthenium(II) hexahydrate (Ru(Bpy); Aldrich) and 40 mM ammonium persulfate (APS; Sigma) were prepared in distilled deionized water. An 18 μl aliquot of 80 μM LMW Aβ_42_ was placed in a PCR tube, followed by 1 μl of Ru(Bpy) and 1 μl of APS. The sample was irradiated (150 W incandescent lamp) for 1 s and the reaction was quenched immediately with 1 M dithiothrietol. Cross-linking reagents were removed by dialysis using 3.5 kDa MWCO Slide-A-Lyzer cassettes (Pierce) against 10 mM sodium phosphate pH 7.4. More than 5 changes of buffer were completed, and protein purity and concentration were confirmed with optical absorption spectroscopy and SDS-PAGE.

For preparation of fibrillar Aβ_42_, LMW Aβ_42_ was prepared as described above, and the peptide preparation was incubated at 100 μM with orbital shaking at 37°C until fibril formation occurred, approximately 2 weeks. The presence of fibrils was confirmed by electron microscopy. We used a previously designed “scrambled” Aβ_42_ peptide that: (1) had an amino acid composition identical to that of wild type Aβ_42_, and (2) did not display amphipathicity (as does the wild type peptide). We accomplished this by using word scrambler software (for example: https://www.wordunscrambler.net/word-scrambler.aspx) to randomly permute the Aβ amino acid sequence. We then used Kyte-Doolittle analysis (88) to determine the hydropathy profiles of the permuted sequences. This sequence met the 2 criteria for scrambled Aβ_42_: YHAGVDKEVVFDEGAGAEHGLAQK -IVRGFGVSDVSMIHINLF.

Both WT and scrambled Aβ_42_ were synthesized using 9-fluorenylmethoxycarbonyl (Fmoc) chemistry, purified by reverse phase-high performance liquid chromatography, and characterized by mass spectrometry and amino acid analysis, as described previously (89). Quantitative amino acid analysis and mass spectrometry yielded the expected compositions and molecular weights for each peptide. Purified peptides were stored as lyophilizates at −20°C. During fibril assembly studies, scrambled Aβ_42_ has been shown to maintain a statistical coil secondary structure, bind thioflavin T poorly, and exhibit oligomerization characteristics distinct from those of wild type Aβ_42_ (90). Scrambled Aβ_42_ was dissolved in 10% (v/v) 60 mM NaOH and 90% (v/v) 10 mM phosphate buffer, pH 7.4, at a concentration of 1 mg/ml.

### Transmission Electron Microscopy

Ten microliters of Aβ were spotted onto 400 mesh carbon-coated Formvar grids (Electron Microscopy Sciences) and incubated for 2 min. Each grid was then negatively stained with 1% (v/v) filtered (0.2 μm) uranyl acetate (Ted Pella), which was immediately wicked off. Electron microcopy analysis was performed using JEOL 1200 EX at 80 KV (91).

All other experimental protocols, including genotyping, GA immunization, isolation and adoptive transfer of bone-marrow CD115^+^ monocytes, quantification and stereological counting, and Barnes maze behavioral tests were previously described (63).

### Statistics

GraphPad Prism 5.0b (GraphPad Software) was used to analyze the data. A comparison of three or more groups was performed using two-way or one-way ANOVA followed by the Tukey's or Bonferonni's (92) *post hoc* multiple comparison test for paired groups. Two-group comparisons were analyzed using two-tailed paired *t*-tests (StatPlus). Correlation analyses were performed using the Pearson's coefficient (*r*) tests (Prism). Results are expressed as mean ± standard error of the mean (s.e.m.). A *p* value less than 0.05 was considered significant.

## Results

### Excitatory Synapses Are More Vulnerable to Aβ_42_ Oligomers Than Fibrils

**Figure 1:**
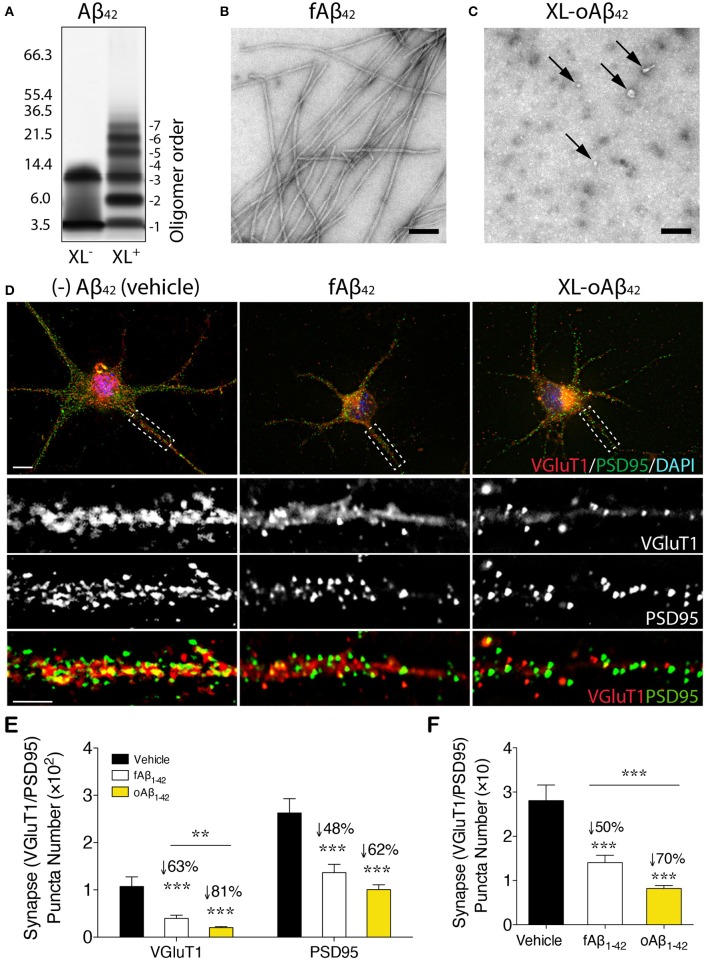
Impact of defined and stabilized Aβ_42_ oligomers vs. purified fibrils on synaptic integrity in primary cortical neurons. **(A)** SDS-PAGE image of cross-linked (XL^+^) and non-XL (XL^−^) Aβ_42_ oligomers. **(B)** Electron microscopy (EM) of preformed fibrillar (f)Aβ_42_ and **(C)** XL-oAβ_42_, consisting of a defined distribution of covalently linked oligomers that were characterized by negative EM stain. Spherical (arrows) and amorphous structures were observed for XL-oAβ_42_, whereas typical amyloid fibrils were seen in fAβ_42_. **(D)** Representative high-resolution images of pre-VGluT1 and post-PSD95 synapses in primary cortical neurons, treated with no (–)Aβ_42_ (vehicle), fAβ_42_ or XL-oAβ_42_. Areas within dashed boxes are magnified below. **(E,F)** Quantification of pre- and postsynapses in postnatal day 1 (P1) mouse primary cortical neurons. **(E)** VGluT1- and PSD95-immunoreactive area per neuron and **(F)** colocalized VGluT1/PSD95 synaptic puncta number in P1 cortical neurons treated with vehicle, fAβ_42_ or XL-oAβ_42_. ^***^P < 0.001, fAβ_42_ vs. vehicle or XL-oAβ_42_ vs. vehicle; ^**^P < 0.01, fAβ_42_ vs. XL-oAβ_42_, by one-way ANOVA and Tukey's post-test. Data expressed as mean ± s.e.m.; *n* = 32 fields analyzed from 2 independent experiments; Scale bars = 100 nm **(B,C)** and 5 μm **(D)**.

To assess the effect of Aβ_42_ oligomers vs. fibrils on synaptic integrity and neuronal structure, postnatal day 1 (P1) primary cortical neurons (CN) were incubated for 12 h with 100 nM of either defined and stabilized oligomers (XL-oAβ_42_), preformed fibrils (fAβ_42_), or non-aggregated scrambled Aβ control peptides (sAβ_42_)(90), compared to medium alone without Aβ (vehicle; Figures 1A–D). The pure populations of Aβ_42_ fibrils and stabilized low-n oligomers were verified by SDS-PAGE gel and transmission electron microscopy (Figures 1A–C). We observed no statistical difference in synaptic puncta number [analyzed by colocalized presynaptic VGluT1 (vesicular glutamate transporter 1) and PSD95 (postsynaptic density protein 95)] or neurite length between vehicle and sAβ_42_ groups (Supplementary Figures 1A,B). There was also no significant difference between CN co-cultured for 24 h with BM-derived MΦ for 24 h supplemented with either medium alone or sAβ_42_ (Supplementary Figures 1A,B). Interestingly, there was a small but significant increase in VGluT1/PSD95 synaptic number in cortical neurons following 24 h co-culture with GA-treated MΦ (Supplementary Figure 1A). While sAβ_42_ did not induce loss of synapses or neuritic length retraction in CN, incubation with fAβ_42_, and moreover, with XL-oAβ_42_ (Figure 1D), decreased presynaptic VGluT1^+^ number by 63 and 81%, respectively, as compared to vehicle (Figure 1E; *P* < 0.001). Similarly, but to a lesser extent, postsynaptic PSD95^+^ puncta numbers were significantly reduced by 48 and 62% after exposure to fAβ_42_ or XL-oAβ_42_, respectively (Figure 1E; *P* < 0.001). Analysis of co-localized VGluT1 and PSD95 puncta number indicated a 50 and 70% reduction in synaptic density after 12 h exposure to fAβ_42_ or XL-oAβ_42_, respectively (Figure 1F; *P* < 0.001). Importantly, cortical excitatory synapses were substantially more vulnerable to low-n Aβ_42_ oligomers than to their fibril counterparts (Figure 1F; *P* < 0.001).

### Spontaneous Neuronal Hyperactivity and Altered Spike Waveforms Induced by Aβ_42_ Oligomers

**Figure 2:**
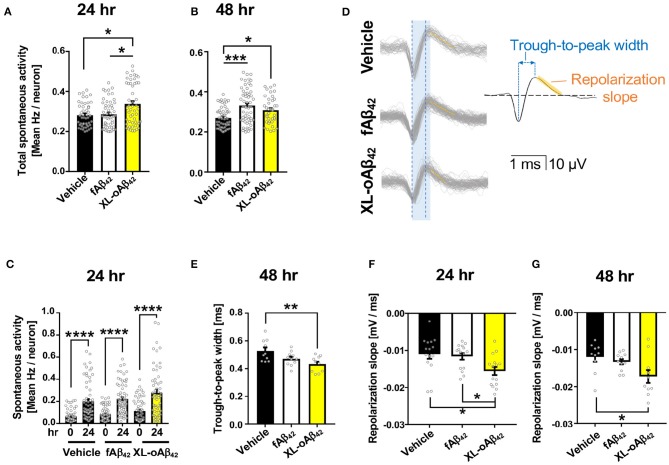
Oligomeric Aβ_42_ assemblies cause neuronal hyperactivity and altered extracellular spike waveforms. Microelectrode array recordings of spontaneous activity in P1 primary cortical neurons measured 24 and 48 h after addition of vehicle, 100 nM of fAβ_42_, or of XL-oAβ_42_. **(A,B)** Mean neuronal frequency of active neurons showing spontaneous activity during 5 min recordings. Neurons analyzed met a minimum threshold of 0.2 Hz firing rate. 24 h, ^*^P < 0.05, vs. Vehicle or fAβ_42_; 48 h, ^*^P < 0.05, ^***^P < 0.001, vs. Vehicle; Kruskal-Wallis analysis by Dunn's test. **(C)** Comparison of spontaneous activity measured from the same individual neuron populations over time, from untreated (time 0) to 24 h incubation with vehicle, fAβ_42_, or XL-oAβ_42_. ^****^P < 0.0001, vs. time 0; Kruskal-Wallis analysis by Dunn's test. **(D)** Representative extracellular waveforms from each condition (gray = overlays of multiple waveforms from a single neuron; black = fitted mean waveform for analysis), identifying the trough-to-peak width (blue) and repolarization slope (orange). Shaded blue box indicates trough-to-peak width observed in control wave forms, dotted blue lines indicate measurement in XL-oAβ_42_. Orange hashed lines indicate mean repolarization slope of each wave form. **(E)** Quantification of trough-to-peak width in P1 cortical neurons incubated with fAβ_42_ or XL-oAβ_42_ for 48 h. ^**^P < 0.01 vs. Vehicle 48 h. **(F–G)** Quantification of repolarization slope in P1 neurons in each condition. ^*^P < 0.05 vs. Vehicle 24 and 48 h or fAβ_42_ 24 h, respectively. one-way ANOVA and Tukey's post-test. Data expressed as mean ± s.e.m., with individual data point from 5 independent experiments.

To examine the functional consequences of exposing neurons to Aβ_42_ oligomers vs. fibrils, we recorded the spontaneous extracellular activity of P1 cortical neurons using Microelectrode arrays (MEA) after incubation with vehicle, fAβ_42_, or XL-oAβ_42_ for either 24 or 48 h (Figures 2A–G and Supplementary Figures 2A–D). We first compared the spontaneous activity levels across all neurons with a level of activity of at least 0.2 Hz. We found that after incubation with Aβ_42_ oligomers for 24 h, spontaneous activity levels were significantly larger compared to incubation with vehicle or fibrils (Figure 2A; *P* < 0.05), whereas activity levels for incubation with fibrils was not significantly different from incubation with vehicle (Figure 2A; *P* < 0.05). After 48 h, neurons incubated with either fAβ_42_ or XL-oAβ_42_ had significantly elevated levels of spontaneous activity compared to vehicle (Figure 2B; *P* < 0.001–0.05). This shows that incubation with Aβ_42_ oligomers is accompanied by hyperactivity as early as 24 h. This was also true when examining all neurons regardless of spontaneous activity levels and when only included neurons recorded at both time points (see Supplementary Figures 2A–C).

Did the presence of oligomers change the biophysical properties of neurons? To address this question, we next assessed whether the extracellular waveforms of the recorded action potentials differed between neurons exposed to fibrillar and oligomeric forms (Figures 2D–G and Supplementary Figure 2D). We found that two critical features of the extracellular waveform changed after incubation with oligomeric Aβ_42_. First, the trough-to-peak width of waveforms (Figure 2D) recorded from neurons incubated with oligomeric Aβ_42_ was significantly decreased relative to controls after 48 h (Figure 2E; *P* < 0.01); this effect was not apparent after 24 h (Supplementary Figure 1D). Second, the repolarization slope significantly decreased for neurons incubated with Aβ_42_ oligomers, but not fibrils, relative to controls after 24 h (Figure 2F; *P* = 0.0103), an effect which persisted through 48 h (Figure 2G; *P* < 0.05). Together, this shows that incubation with Aβ_42_ oligomers was accompanied by spontaneous neuronal hyperactivity and altered extracellular waveforms.

### Activated Macrophages Effectively Prevent Synaptic Loss and Neuritic Arborization Retraction Caused by Aβ_42_ Oligomers

**Figure 3:**
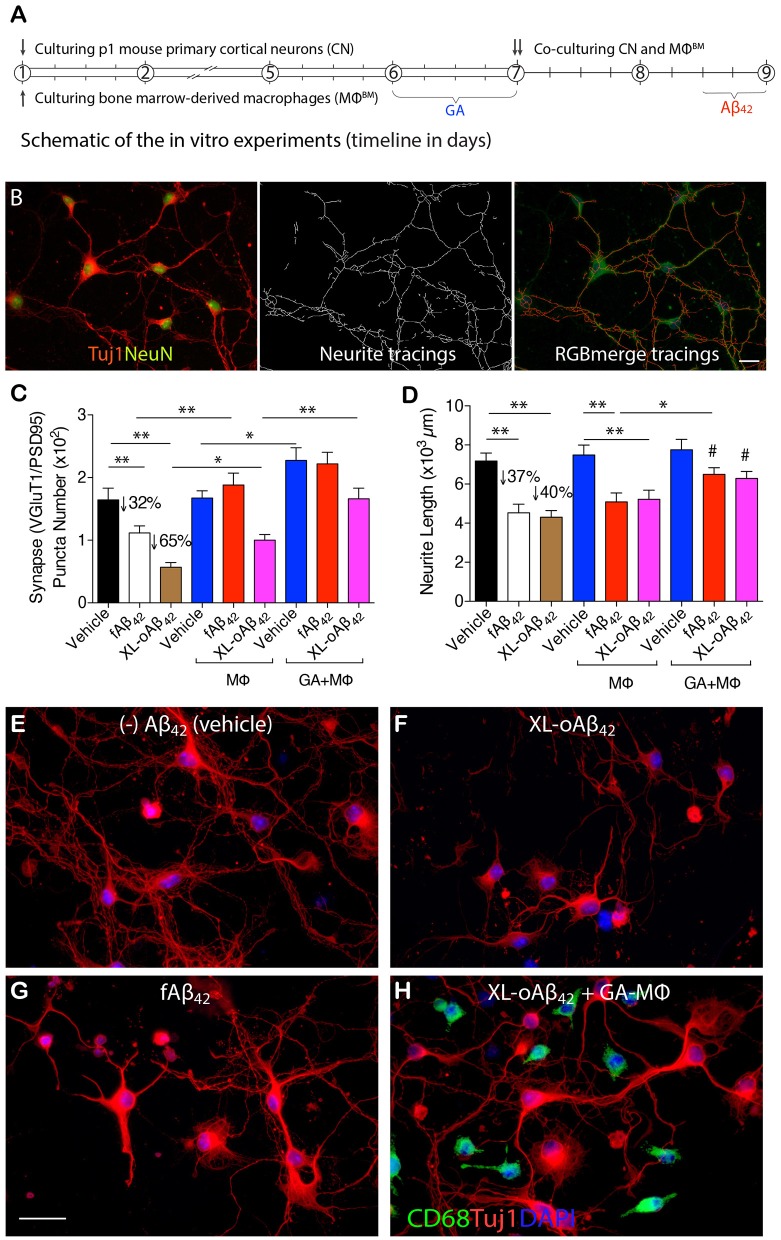
Activated MΦ effectively protect against oligomeric Aβ_42_-induced synaptic and neuritic arborization loss in primary cortical neurons. **(A)** Schematic of the *in vitro* experiments (timeline in days). P1 cortical neurons (treated with 100 nM XL-oAβ_42_, fAβ_42_, or vehicle for 12 h, respectively), bone marrow-derived MΦ (MΦ^BM^), and GA-activated MΦ^BM^ (GA-MΦ) were cultured for 9 d. **(B)** Representative microphotographs of P1 neurons labeled with anti-Tuj1 and -NeuN serum (left), neuritic tracings with NeuriteTracer (83) (middle), and RGB merge tracings (right). Scale bar represents 20 μm. **(C)** Quantification of colocalized VGluT1/PSD95 synaptic puncta number in P1 neurons incubated with fAβ_42_, XL-oAβ_42_, or vehicle, and P1 neurons co-cultured with MΦ or with GA-MΦ. Note that fAβ_42_ and XL-oAβ_42_ both reduced the VGluT1/PSD95 synaptic density that was significantly preserved by co-culturing with MΦ. This effect was enhanced by co-culturing with GA-MΦ. **(D)** Quantification of neuritic length of P1 neurons incubated with fAβ_42_, XL-oAβ_42_, or vehicle, and P1 neurons co-cultured with MΦ or with GA-MΦ. Note that co-culturing with GA-MΦ significantly prevented decreases in neuritic length from fAβ_42_ or XL-oAβ_42_. Data expressed as mean ± s.e.m.; *n* = 48 fields analyzed from 3 independent experiments; ^*^P < 0.05, ^**^P < 0.01, comparisons as indicated by lines; ^#^*P* < 0.05, vs. fAβ_42_ or XL-oAβ_42_ alone (no MΦ), by one-way ANOVA and Tukey's post-test. **(E-H)** Representative microphotographs of primary P1 neurons incubated with **(E)** vehicle, **(F)** XL-oAβ_42_, **(G)** fAβ_42_, and **(H)** co-cultured with GA-MΦ + XL-oAβ_42_. Scale bar = 20 μm.

To determine whether naïve BM-derived (MΦ^BM^) or GA-activated macrophages (GA-MΦ) can confer neuroprotection, P1 CN were co-cultured with MΦ^BM^ or GA-MΦ in the presence of fAβ_42_ or XL-oAβ_42_ (Figure 3; experimental scheme and neurite tracing quantification method are shown in Figures 3A,B). Substantial synaptic loss and neuritic retraction were observed, especially after exposure to Aβ_42_ oligomers (Figures 3C,D,F,G vs. Figure 3E; *P* < 0.01; 32 and 65% VGluT1/PSD95-synaptic loss in response to fibrils and oligomers, respectively). Importantly, reductions in pre- and postsynaptic puncta number (and area) after exposure to fAβ_42_ were fully restored when co-cultured with MΦ^BM^ or GA-MΦ (Figure 3C and Supplementary Figures 3A, B; *P* < 0.01), suggesting that MΦ have the capacity to effectively protect synapses against Aβ_42_ fibril toxicity within this time frame. However, untreated MΦ had limited ability to protect synapses against damage induced by Aβ_42_ oligomers (Figure 3C; *P* < 0.05), although importantly, GA activation of MΦ promoted full protection also against these highly synaptotoxic oligomers (Figure 3C and Supplementary Figures 3A,B; *P* < 0.01). While minimal to no protection against neuritic arborization loss was observed following co-culturing cortical neurons with naïve MΦ in the presence of either fAβ_42_ or XL-oAβ_42_ (Figure 3D), significant protection to neuronal dendritic structure was obtained with GA-activated MΦ, as measured by neurite process lengths (Figure 3D; *P* < 0.05). Interestingly, even in the absence of Aβ, co-cultures of cortical neurons with GA-MΦ vs. naïve MΦ had a significant increase in synaptic density (Figure 3C; *P* < 0.05). Thus, in primary cortical neurons, GA-activated MΦ effectively prevented loss of synapses and defects in arborization of neurite processes induced by both Aβ_42_ fibrils and oligomers.

### MMP-9/Extracellular Degradation Is Predominant for Macrophage Clearance of Aβ_42_ Oligomers

**Figure 4:**
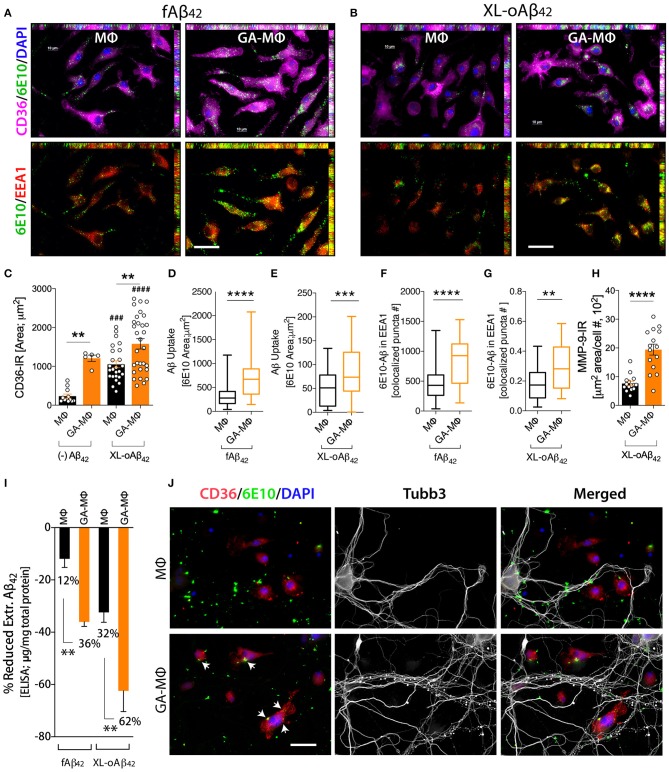
Induced oligomeric Aβ_42_ clearance via GA activation of CD36/EEA1-intra- and MMP-9-extracellular mechanisms in MΦ, leading to neuronal preservation. **(A–G)** GA-activated (24 h) or naïve MΦ were exposed to 100 nM Aβ_42_ assemblies for 30 min, and co-labeled for Aβ (6E10, green), type B scavenger receptor CD36 (magenta), and early endosomal marker (EEA1, red). Cells were counterstained with DAPI (blue). Representative micrographs demonstrate elevated surface CD36 expression, intracellular Aβ, and Aβ colocalized within EEA1 endosomes in GA-MΦ vs. control MΦ, 30 min fAβ_42_ **(A)** and XL-oAβ_42_ **(B)** uptake assays. **(C)** Quantitative immunoreactive analysis of CD36 signals showed a substantial increase in CD36 in GA-MΦ. Basal levels of CD36 expression without Aβ_42_ were higher in GA-MΦ vs. control MΦ. This increase maintained its significance after exposure to XL-oAβ_42_. Regardless of GA effects, the presence of oligomeric Aβ_42_ substantially increased CD36 levels in both control MΦ and GA-MΦ. **(D,E)** Quantitative analysis of intracellular Aβ in MΦ following 30 min fAβ_42_ **(D)** and XL-oAβ_42_ **(E)**. **(F,G)** Quantitative analysis of fAβ_42_ **(F)** and XL-oAβ_42_ **(G)** within EEA1-positive vesicles in early endosomes in GA-MΦ vs. control MΦ. Co-labeled 6E10/EEA1- immunoreactive puncta number per MΦ is displayed. **(H)** Increased MMP-9 signal in GA-MΦ after exposure to XL-oAβ_42_. **(I)** ELISA quantification of extracellular Aβ_42_ after 24 h incubation with fAβ_42_ or XL-oAβ_42_ compared to 30 min basal peptide levels. **(J)** Representative microphotographs of co-cultured P1 neurons and MΦ exposed to XL-oAβ_42_ for 24 h, stained with CD36, 6E10, Tubb3, and DAPI. P1 cortical neurons co-cultured with GA-MΦ vs. control MΦ exhibit increased neuritic outgrowth density with increased CD36-mediated Aβ uptake (arrows) and reduced extracellular Aβ_42_. Data expressed as mean ± s.e.m., with individual data point; *n* = 48 fields analyzed from 3 independent experiments; immunoreactive areas are normalized by cell number; ^**^P < 0.01, ^***^P < 0.001, ^****^P < 0.0001, GA-MΦ vs. MΦ with fAβ_42_ or XL-oAβ_42_, and ^*###*^*P* < 0.001, ^*####*^*P* < 0.0001 for comparisons to MΦ without Aβ [(–) Aβ_42_], by one-way ANOVA with Tukey's test or two-tailed student *t*-test. Scale bar = 20 μm.

To investigate how macrophages, including GA-activated macrophages, facilitate clearance of Aβ_42_ oligomers vs. fibrils and potentially increase synaptic protection, we characterized MΦ phenotypes, with and without GA stimulation, in response to the same quantities of XL-oAβ_42_ and pre-formed fAβ_42_ (100 nM; Figure 4). First, to assess surface recognition and intracellular uptake of Aβ_42_ assemblies, MΦ were incubated with XL-oAβ_42_ or fAβ_42_ for 30 min and labeled for Aβ (6E10), early endosome antigen 1 (EEA1), and class B scavenger receptor CD36 or class A type 1 scavenger receptor SCARA1 (encoded by macrophage scavenger receptor 1 – *MSR1* gene). There was a notable increase in CD36 expression, intracellular o/fAβ_42_, and o/fAβ_42_ colocalized within EEA1 endosomes in GA-MΦ vs. naïve MΦ (Figures 4A, **B**). A quantitative analysis of scavenger receptors indicated a significant increase in CD36 expression (Figure 4C; *P* < 0.01; Supplementary Figure 4A; *P* < 0.05) in GA-MΦ compared to untreated MΦ, but not in SCARA1 for XL-oAβ_42_ (Supplementary Figure 4B). Regardless of GA, exposure to Aβ_42_ oligomers substantially upregulated surface expression of CD36 in MΦ (Figure 4C; *P* < 0.001). GA-MΦ exhibited significantly more intracellular uptake of both fAβ_42_ and XL-oAβ_42_ vs. naïve MΦ (Figures 4D,E; *P* < 0.0001 and *P* < 0.001, respectively). Both naïve and GA-MΦ were on average six times less effective in intracellular uptake of oligomeric vs. fibrillar Aβ_42_ (Figure 4D vs. Figure 4E). Strikingly, as compared with fibrils, Aβ_42_ oligomers were approximately 2,500 times less targeted into early endosomes (Figure 4F vs. Figure 4G), as measured by 6E10^+^-Aβ puncta number colocalized in EEA1^+^ vesicles after 30 min phagocytosis assay. Hence, these data suggest that while intracellular Aβ_42_ fibrils are predominantly targeted to the endosomal-lysosomal proteolysis pathway, intracellular oligomers mostly escape these proteolytic vesicles in MΦ. Nonetheless, GA-MΦ significantly increased the presence of both Aβ_42_ fibrils and oligomers in EEA1^+^ endosomes (Figures 4F,G; *P* < 0.01–0.0001).

Next, we evaluated the extent to which MΦ^BM^ degrade Aβ_42_ oligomers as compared to fibrillar forms in the extracellular space. To this end, we first assessed the expression of matrix metallopeptidase 9 (MMP-9)—an Aβ-degrading enzyme—by analysis of MMP-9^+^-immunoreactive area in MΦ (Figure 4H). Then, we determined Aβ_1−42_ concentrations in supernatant of MΦ following incubation with 100nM of either fAβ_42_ or XL-oAβ_42_, using a highly sensitive ELISA assay (Figure 4I). We previously showed that after exposure to Aβ_42_ fibrils, GA-MΦ exhibited a significant increase in MMP-9 levels (63, 70). Yet, the response to oligomers has not been reported. Here, we found that MMP-9 expression in GA-MΦ was *markedly* increased after incubation with XL-oAβ_42_ (Figure 4H; *P* < 0.0001). Examination of extracellular degradation as a function of Aβ_42_ conformation indicated significant decreases in concentration of Aβ_42_ in the media of GA-MΦ vs. naïve MΦ after 24 h incubation with either fAβ_42_ or XL-oAβ_42_ (Figure 4I; 36 or 62% reduction, respectively, *P* < 0.01). Likewise, the substantial reduction in fAβ_42_ and XL-oAβ_42_ concentrations in the extracellular media of GA-treated vs. untreated MΦ can be seen after 30 min of incubation with these Aβ species (Supplementary Figures 4C,D; *P* < 0.001). Notably, at the two incubation time points (30 min and 24 h incubation), Aβ_42_ oligomers were primarily degraded in the extracellular rather than the intracellular space of macrophages and were considerably more accessible for extracellular degradation than their fibril counterparts (Figure 4I; 32% vs. 12 and 62% vs. 36% for MΦ and GA-MΦ, respectively; Supplementary Figures 4C,D; *P* < 0.001). Representative microscopic images in Figure 4J panel illustrate the increase in oAβ_42_ uptake and CD36 expression by GA-activated MΦ, along with a decrease in extracellular or synaptic-bound oAβ_42_, which resulted in synaptic and neuritic outgrowth preservation in primary cortical neurons. Overall, these data suggest that GA induces CD36/EEA1-mediated macrophage clearance, and moreover, MMP-9/extracellular degradation of synaptotoxic Aβ_42_ oligomers, ultimately protecting synapses, their dendritic structure, and neural function.

### Severe Loss of Excitatory Pre- and Postsynaptic Terminals in the Entorhinal Cortex and Hippocampus of ADtg Mice

**Figure 5:**
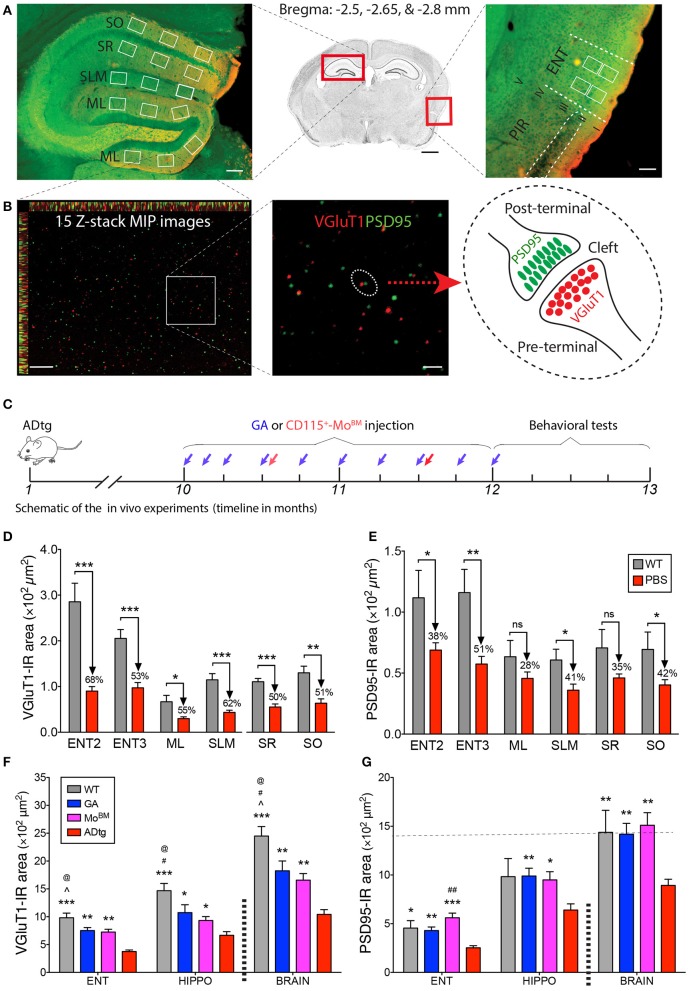
Cortical and hippocampal synaptic rescue by CD115^+^-monocyte blood enrichment and GA immunization in ADtg mice. **(A,B)** VGluT1/PSD95 synaptic quantification scheme in entorhinal cortex (ENT) and hippocampus (HIPPO). **(A)** (Middle) Representative crystal violet stained image of a coronal brain section at Bregma −2.65 mm. Scale bar = 1 mm. (Left) Representative microimage of HIPPO stained with the presynaptic (VGluT1, red) and postsynaptic (PSD95, green) markers. Note that 3 fields are chosen in each of the lateral and medial blade molecular layers (ML) of dentate gyrus (DG), as well as in the stratum lacunosum-moleculare (SLM), stratum radiatum (SR), and stratum oriens (SO) of CA1. (Right) Representative microphotograph of ENT immunolabled with the same pre- and postsynaptic markers. Note that ENT adjacent to piriform cortex (PIR) and 2 same fields are precisely selected from layers 2 and 3. Scale bar = 100 μm. **(B)** Representative microphotographs from the lateral blade ML immunostained with the same synaptic markers. Each high-magnification maximum intensity projection (MIP) image contains 15 Z-stack optical scanning (left). Scale bar = 10 μm. (Middle) Magnification for visualizing the proximity of pre- and postsynaptic signals. Scale bar = 2 μm. (Right) Schematic representation of pre- and post-terminal synapse. **(C)** Schematic of the *in vivo* experiments, where 10-month-old ADtg mice (all males) were injected with weekly s.c. GA immunization or monthly i.v. CD115^+^-Mo^BM^ injections for a duration of 2 months. Control groups were either naïve WT mice or monthly i.v. PBS injected ADtg mice. At the completion of the experiment, mice underwent behavioral testing, were euthanized, and brains were collected for analyses. **(D,E)** Quantification of presynaptic VGluT1 **(D)** and postsynaptic PSD95 **(E)** areas in ENT2/3 and hippocampal substructures of 13-month-old ADtg mice vs. age-matched WT littermates. **(F,G)** Quantification of VGluT1 **(F)** and PSD95 **(G)** immunoreactive areas in the entorhinal cortex, hippocampus, and whole brain of WT, GA-immunized, Mo^BM^-injected, and PBS-control mice. Data expressed as mean ± s.e.m.; *n* = 6 mice per group; ^*^P < 0.05, ^**^P < 0.01, ^***^P < 0.001, and ^#^*P* < 0.001, ^*##*^*P* < 0.0001, compared to ADtg control; @*P* < 0.05 compared to Mo^BM^, ^∧^*P* < 0.05 compared to GA, one-way ANOVA with Tukey's test.

Given the crucial role of entorhinal cortex layers 2 and 3 in connecting brain cortical regions to the hippocampus, we sought to analyze pre- and postsynaptic VGluT1 and PSD95 biomarkers in substructures of the entorhinal cortex (ENT) and hippocampus (HIPPO) of 10- and 13-month-old symptomatic ADtg vs. wild type (WT) mice (Figures 5A–**E**; Supplementary Figures 5–8). Due to lack of differences between the lateral (ML-L) and the medial (ML-M) blade molecular layers in pre- and postsynaptic areas across all experimental groups (Supplementary Figure 5), both regions were combined in our analyses (Figures 5D,E). Ten-month-old ADtg mice already exhibited significant pre- and postsynaptic losses in the molecular layers (ML) of the dentate gyrus (DG) and the stratum lacunosum-moleculare (SLM), but not in the stratum oriens (SO) nor in the stratum radiatum (SR) of the cornu ammonis 1 (CA1; Supplementary Figure 6). At 13 months, ADtg mice exhibited 50-62% presynaptic VGluT1-immunoreactive (IR) area loss in all hippocampal regions as compared to WT mice (Figure 5D; *P* < 0.05–0.001). Similarly, analyses of postsynaptic PSD95-IR areas in 13-month-old ADtg mice vs. WT littermates indicated significant reductions by 28–42% in all hippocampal regions (Figure 5E; *P* < 0.05). In ENT layers 2 and 3, 68 and 53% of presynaptic VGluT1-positive areas were lost (Figure 5D; *P* < 0.001), while 38 and 51% of postsynaptic PSD95 signals were reduced (Figure 5E; *P* < 0.05 and *P* < 0.01) in ADtg mice compared to WT mice, respectively. Overall, our data demonstrate a progressive and considerable loss of VGLUT/PSD95 synaptic density, while the presynaptic VGluT1 terminals appear more vulnerable to Aβ accumulation in the 13-month ADtg animals.

### Synaptic Rescue Following Enhanced Cerebral Recruitment of Macrophages via Immunomodulation Therapy in ADtg Mice

To better understand the role of peripheral Mo/MΦ in preservation of excitatory synapses within AD-relevant cortical and hippocampal regions, we analyzed both pre- and postsynaptic density in substructures of ENT and HIPPO following weekly s.c. injections of GA immunization or monthly i.v. injections of CD115^+^ Mo^BM^ into the peripheral blood (schematic of *in vivo* experiment in Figure 5C). Rescue pre- and postsynaptic density was noted in ENT2/3 by GA immunization or blood enrichment with Mo^BM^ in ADtg mice (Figures 5F,G, *P* < 0.05–0.001; Supplementary Figure 7, *P* < 0.05–0.01). Similarly, GA led to rescue of pre-VGluT1 and post-PSD95 synapses in HIPPO across various ML, SLM, SR, and SO subregions (Figures 5F,G, *P* < 0.05–0.01; Supplementary Figure 8). Notably, throughout the analyzed brain regions, both immunomodulation approaches rescued postsynaptic density (i.e., PSD95-IR area) in symptomatic ADtg mice to levels similar or higher than those observed in WT mice (Figure 5G; Supplementary Figures 7B, 8B). No cumulative effect was noted between a group of ADtg mice treated with both GA and Mo^BM^ (combined treatment) and groups of mice treated with GA or Mo^BM^ alone (data not shown). Taken together, our *in vitro* and *in vivo* data suggest that immunomodulation approaches that deliver macrophages to reduce pathogenic Aβ_42_ burden at synaptic clefts substantially preserve pre- and postsynaptic terminals.

To identify confounders that potentially predict excitatory synaptic loss and contribute to immunotherapy-based synaptic preservation, we carried out multiple Pearson's coefficient (*r*) correlation analyses in the 13-month-old mouse cohort (Supplementary Figure 9). Specifically, we explored possible relationships between synaptic integrity and parameters of disease stage (cognitive function in Barnes maze test, astrogliosis, and amyloidosis) and therapeutic response (cerebral MΦ recruitment and phenotype). A strong linear association was observed between cerebral astrocytosis (GFAP^+^ reactive astrocyte area) and impaired cognitive function, as measured by error count on day 7 (long-term memory retention) of Barnes maze test (Supplementary Figure 9A; *P* = 0.0187, Pearson's *r* = 0.60). Similar correlations were found between hippocampal and cortical astrogliosis and other parameters of cognitive deficit in the Barnes maze test (not shown), suggesting that astrogliosis is a strong predictor of cognitive decline. Comparable to these observations, tight correlations were detected between cortical astrocytosis and postsynaptic loss (Supplementary Figures 9B–**D**; *P* = 0.028–0.007 and Pearson's *r* = −0.55 to −0.61). These data show an association between astrogliosis and cognitive outcome.

In relation to Aβ-plaque pathology, both cortical and hippocampal regions further revealed close inverse associations with postsynaptic density (Supplementary Figures 9E–H; *P* = 0.011–0.002 and Pearson's *r* = −0.57 to −0.70). Similar correlations were also observed for presynaptic areas (not shown). These data suggest that cerebral Aβ-plaque burden are important indicators of synaptic loss in this murine model. We then established the relationship between excitatory synaptic integrity and cognition, showing significant associations between hippocampal and cortical synaptic density and cognitive function (Supplementary Figures 9I,J; *P* = 0.015–0.009 and Pearson's *r* = −0.45 to −0.53). The latter cognitive performance was determined by escape latency times in the learning and memory reversal phase on day 9 of the Barnes maze test. These data suggest that synaptic integrity is tightly associated with cognitive function, and that both astrogliosis and amyloidosis adversely affect it.

We previously showed that effective immunomodulation approaches in ADtg mice, such as GA immunization or blood enrichment with Mo^BM^, reduced Aβ-plaque pathology and preserved cognition (50, 52, 63, 70). These therapeutic effects were achieved via cerebral recruitment of Iba-1^+^/CD115^+^/CD45^hi^ Mo/MΦ that expressed high levels of osteopontin (OPN; encoded by the gene secreted phosphoprotein 1 or SPP1) (63, 70). OPN/SPP1 is an immunoregulatory cytokine shown to both promote synaptogenesis (93–95) and be crucial for MΦ-mediated Aβ_42_ clearance (70). Significant and strong associations were observed between cerebral and hippocampal Iba-1^+^/CD45^hi^ Mo/MΦ (Mo/MΦ cell count) and cognitive function, as measured by latency times and error counts on day 9 of the Barnes maze test (Supplementary Figures 9K,L; *P* = 0.0472–0.011 and Pearson's *r* = −0.64 – −0.76). We further found a significant and robust association between OPN and postsynaptic but not presynaptic density areas (Supplementary Figures 9M,N; *P* = 0.0133 and Pearson's *r* = 0.69). These analyses suggest that increased Mo/MΦ-mediated OPN/SPP1 presence in the brain is linked with synaptic integrity and cognitive preservation.

## Discussion

This study supports the conclusion that activated MΦ^BM^ can reduce Aβ_42_ oligomers and protect synaptic integrity and neuronal structure. Initially, we determined the impact of defined and stabilized populations of soluble Aβ_42_ oligomers on excitatory synapses, neuritic arborization, and neuronal function as compared to pure fibrils. We indicated that synaptotoxicity was not elicited by non-aggregating sAβ_42_ peptides but was dependent on their conformation state; cortical neurons were far more vulnerable to Aβ_42_ oligomers than to fibrils. Aβ_42_ oligomers caused severe synaptic loss, neurite arborization retraction, and hyperactivity, as well as changes in two critical features of the extracellular waveform in cortical neurons. We further found that MΦ^BM^ effectively protect against synaptic loss and neurite retraction caused by Aβ_42_ fibrils yet provide insufficient protection against oligomers. Importantly, MΦ activation with glatiramer acetate (GA, an FDA-approved drug) fully protected neurons from detrimental effects triggered by both oligomers and fibrils. We identified distinct MΦ-mediated mechanisms for clearance of oligomers as compared to fibrils leading to synaptic protection. While eradication of Aβ_42_ oligomers predominately involves increased extracellular/MMP-9 degradation, fibrils are primarily eliminated via CD36/EEA1^+^-endosomal intracellular proteolysis. Strikingly, both pathways are boosted in MΦ stimulated with GA. Finally, our *in vivo* studies corroborated the *in vitro* findings, demonstrating that loss of cortical and hippocampal excitatory synapses in transgenic mouse models of AD is reversed by increased cerebral recruitment of Mo-derived MΦ, achieved via GA immunomodulation or peripheral-blood enrichment of BM-derived monocytes. Overall, these findings should guide future therapeutic interventions for AD to boost MΦ-mediated clearance of synaptotoxic Aβ_42_ oligomers and preserve synapses and cognition.

Our data in mouse primary cortical neurons provide evidence to support the notion that Aβ_42_ oligomers pose a greater threat to the neuronal network than do fibrils. These results align with previous studies reporting that Aβ_42_ oligomers impair neuronal plasticity and activation of excitatory mGluR hippocampal neurons, as well as exhibit direct synaptotoxic effects leading to cognitive impairments (8, 13, 15, 23–29, 96). As compared to postsynaptic terminals, presynaptic terminals displayed greater vulnerability to Aβ_42_ assemblies in both *in vivo* and *in vitro* models. We found that compared to fibrils, stabilized Aβ_42_ oligomers caused greater synaptic loss and neuritic retraction along with hyperactivity and altered action potential shape in primary cortical neurons. Due to the massive reduction in synapses (~70%) observed in response to Aβ_42_ oligomers as early as 12 h after incubation, a corresponding reduction in neuronal activity was anticipated. To the contrary, however, we found elevated levels of spontaneous activity after incubation with oligomers. These observations are in accordance with previous studies in murine models of AD that showed increased neuronal excitability near Aβ plaques or in Aβ-depositing mouse brains, using Ca^2+^ imaging and *in vivo* intracellular recording (97, 98). Combined with the modified action potential shape we documented, this suggests that oligomeric Aβ_42_ directly affects the biophysical (intrinsic) properties of neurons, possibly via altered ion channel densities, in addition to its effects on synapses. Because oligomeric Aβ_42_ species are highly metastable and exist in dynamically changing mixtures, the conclusions of previous reports on Aβ_42_ oligomers may have been confounded by the existence of heterogeneous Aβ populations. By using defined and stabilized Aβ_42_ oligomers, this study establishes the precise neuropathological consequences caused by oligomers vs. fibrils and supports therapeutic avenues that clear cerebral Aβ_42_ oligomers to prevent such neuronal and synaptic damage.

One powerful method to clear Aβ_42_ oligomers and protect synapses was revealed in this study. Cortical neurons that were severely impaired by Aβ_42_ oligomers showed partial recovery of pre- and postsynaptic density and neurite arborization when co-cultured with MΦ^BM^, and full recovery with GA-MΦ. While naïve MΦ were able to effectively protect against synaptic and neurite length loss caused by the fibrils, they exhibited poor capacity to prevent oligomeric-induced synaptotoxicity. Yet, GA-MΦ successfully eliminated both fibrils and oligomers, essentially protecting neuronal network integrity despite these adverse conditions. Postsynaptic terminals, which were less vulnerable to Aβ_42_ species, were fully rescued by the presence of GA-MΦ. However, it would be interesting to look at the alteration of inhibitory gephyrin immunosignal expression following the administration of GA or GA-MΦ. We discovered that the EEA1^+^-early endosomal/lysosomal pathway was far more involved in the MΦ-mediated engulfment of fibrils than of oligomers. In contrast, reduction of Aβ_42_ in the extracellular space of MΦ was substantially more efficient for oligomers than for fibrils, potentially through enhanced release and activity of proteolytic enzymes (i.e., MMP-9). These *in vitro* data corroborate our *in vivo* studies in ADtg mice that demonstrated enhanced MMP-9 expression by infiltrating macrophages surrounding cerebral Aβ plaques in GA-immunized animals (63, 70). Notably, GA enhanced both internalization and extracellular pathways in MΦ to break down synaptotoxic Aβ_42_ forms. Recent studies indicated that depleting microglia with colony-stimulating factor 1 receptor (CSF1R) signaling inhibitors could impair formation of the plaques in an AD model (99, 100). Microglial depletion was further found not only to lead to a complete rescue of deficits in proliferation, differentiation and survival of adult hippocampal neural progenitor cells (101), but also significantly reduce ApoE, which is a major constituent of amyloid plaques and promotes their aggregation and deposition (102). It would be interesting to look at changes of synapses, Aβ deposition and cognition when microglial depletion plus GA immunotherapy is applied to this ADtg model. Additional studies are needed to further establish direct structure-function connection between these and other Aβ_42_ forms (e.g., dimers, trimers, etc.), synaptotoxicity, and macrophage-mediated synaptic protection. A limitation of this study is that it does not allow for conclusions regarding potential rescue of neuronal function by macrophages due to lack of electrophysiological studies in macrophages and neurons co-cultures. Yet, since this study clearly demonstrates neuroprotection by morphological (neurite length) and synaptic biomarker measurements, future studies should evaluate whether these are accompanied with functional protection.

In this study, we demonstrated that progressive cortical and hippocampal pre- and postsynaptic loss tightly correlated with increased cerebral Aβ burden in the double-transgenic APP_SWE_/PS1_ΔE9_ murine model. The direct association between pre- and postsynaptic biomarkers and cognitive scores further emphasizes a potential link between Aβ_42_, synaptotoxicity, and cognitive decline. Moreover, the unexpected strong association between synaptic density and GFAP^+^ astrogliosis suggested that beyond direct Aβ toxicity, chronically reactive astrocytes not only fail to protect synapses, but also detrimentally impair them and lead to cognitive decline. Future investigations are warranted to better understand the impact of astrocytes and their phenotype on synaptic and cognitive decline in AD.

Here, we showed that both immunotherapies—GA immunization or blood-enrichment with CD115^+^ monocytes—restored presynaptic and fully rescued postsynaptic density in ADtg mice exhibiting significant synaptic loss. In our previous studies, these immunomodulation approaches have led to increased cerebral infiltration of monocyte-derived macrophages directly involved in Aβ clearance, leading to improved hippocampal-based memory and learning (52, 63). In this study, cognitive function, which directly correlated with synaptic integrity, also strongly associated with cerebral recruitment of (GFP^+^) Iba1^+^/CD45^high^ monocytes and macrophages. These data suggest that MΦ activity in the brain associated with decreased synaptotoxic Aβ levels can enhance synaptic protection and regeneration. One interesting question would be what happens if microglia as residential macrophages are treated with GA. Immunophenotypes of microglia expressed differently in dementia with AD pathology. For example, Iba1 was negatively associated with AD while levels of CD68, MSR-A, and CD64 expression were positively related to the presence of dementia (103). In a previous paper we determined a significant reduction of GFAP expression in the cortex of AD mice following GA or/and GA-MΦ immunotherapy (63), and found GFAP expression in this model was negatively associated with pre-synapse VGluT1. We now further demonstrated that the relationship of GFAP levels vs. post-synapse PSD95 in the cortex of AD mice exhibits the same behavior pattern as that of GFAP vs. VGluT1.

Cerebral clearance of Aβ by innate immune cells has been shown to take place via multiple mechanisms, including phagocytosis and enzymatic degradation (48, 49, 51, 54, 56, 57, 61, 104–106). Increasing evidence over the past decade has established the notion that peripheral MΦ contribute to the phagocytosis of cerebral Aβ plaque, an idea that is further supported by reports of defective Aβ phagocytosis by MΦ isolated from AD patients (52, 53, 58, 60, 63, 104, 107). Several studies corroborating this idea have shown the involvement of scavenger receptors such as CD36 and SCARA-1 in facilitating fibrillar Aβ engulfment by innate immune cells. Rare variants in the *TREM2* gene were also associated with diminished MΦ phagocytosis and increased risk for AD (108–110). Indeed, parallel to the aforementioned investigations, this study's findings indicated elevated levels of surface scavenger receptors (e.g., CD36) in response to not only fibrillar but also oligomeric Aβ_42_, with increased uptake by GA-MΦ. Importantly, the role of MΦ and, moreover, GA-activated MΦ in the clearance of non-fibrillar oligomeric Aβ forms has not been previously studied (111, 112). Enhanced colocalization of oligomers and fibrils in EEA1^+^-vesicles in GA-MΦ imply that these Aβ assemblies are redirected to lysosomal degradation rather than for perinuclear or endoplasmic reticulum pathologic accumulation (113). Therefore, this FDA-approved drug emerges again as a promising modality to safely stimulate phagocytic MΦ and improve ability to resist synaptotoxic Aβ forms associated with AD.

In the absence of Aβ, cortical neurons supplemented with GA-MΦ exhibited elevated levels of synaptic density and neuritic length that were higher than that of the baseline levels detected in cortical neurons alone. These results suggest that increased synaptic density in the presence of GA-MΦ is a result of both Aβ_42_ clearance and upregulation of neurotrophic factors stimulating synaptogenesis, such as insulin-like growth factor 1 (IGF-1), transforming growth factor beta 1 (TGFβ1), and/or OPN/SPP1 (50, 66, 70). In our previous study, we observed that OPN/SPP1 was markedly elevated in MΦ following GA treatment *in vitro* and *in vivo* (70). Here, a strong correlation (Pearson's *r* = 0.7) was found between cerebral OPN/SPP1 levels and postsynaptic density, which can explain increased synaptogenesis in the presence of MΦ^BM^ overexpressing OPN in the brains of immunized ADtg mice (70). In support of these findings, we reported that harnessing monocyte-derived macrophages by GA- or MOG-45D-immunization in ADtg mice enhanced cerebral expression of neurotrophic factors such as IGF-1, TGFβ1, and nuclear transcription factor early growth response 1 (Egr-1) (50, 58, 66), concomitant with recovered hippocampal neurogenesis. Hence, this immunomodulation approach holds great promise to positively affect neural regeneration as well as synaptic and cognitive preservation through combined MΦ activity of eradicating pathogenic Aβ_42_ forms and increasing neurotrophic support.

In conclusion, therapeutic approaches designed to prevent synaptotoxicity or even rescue synapses are becoming more prominent. Our extended data from both *in vitro* and *in vivo* studies may signify that GA-stimulated macrophages confer a potential synaptoprotective phenotype, at least in part through augmented capacity to eliminate highly toxic Aβ_42_ oligomers and induce synaptic preservation and regeneration. Hence, this study encourages the development of a macrophage-based approach as therapeutic intervention for AD.

## Data Availability Statement

The raw data and protocols used to generate datasets for this study are presented in the article text, figures and tables. Supplementary data are also available online. Extended data can be made available upon reasonable request from the corresponding author to any qualified researcher.

## Ethics Statement

The animal study was reviewed and approved by Cedars-Sinai Medical Center Institutional Animal Care and Use Committee (IACUC).

## Author Contributions

MK-H: study conception, design, study supervision, and final approval of the manuscript. SL: major experimental contributor, data acquisition and design. VG, YK, D–TF, JS, AR, and TT: experimental contributors with assistance from MK-H and SL. EH, JS, and DT: preparation of Aβ_42_ assemblies. DD: correlational analysis with guidance from MK-H. SL, VG, and MK-H: data analysis, interpretation, and presentation. MK-H, SL, EH, D–TF, TT, YK, UR, KB, and DT: discussion on intellectual content and manuscript editing. MK-H, SL, EH, VG, D–TF, UR, and DT: manuscript drafting, editing, and revisions.

### Conflict of Interest

The authors declare that the research was conducted in the absence of any commercial or financial relationships that could be construed as a potential conflict of interest.
