# Supplementary material for: Activated Bone Marrow-Derived Macrophages Eradicate Alzheimer's-Related Aβ42 Oligomers and Protect Synapses
Source: Front Immunol. 2020 Jan 31;11:49. doi: 10.3389/fimmu.2020.00049 (PMC7005081; doi:10.3389/fimmu.2020.00049)
Supplement: Supplementary file 1 [file Data_Sheet_1.pdf]

Table S1. Primary and secondary antibodies used in the studies

Antibody (Ab)	*Species*	*Ab type*	*Dilution*	*Source*	*Catalog #*
Primary antibody					
6E10	Mouse	monoclonal	1:100	Covance	SIG-39320
CD36	Rat	monoclonal	1:200	Abcam	ab80080
CD45	Rat	monoclonal	1:25	BD Pharmingen	550539
CD68	Rat	monoclonal	1:100	Abcam	ab53444
EEA1	Rabbit	polyclonal	1:100	MilliporeSigma	07-1820
GFAP	Rabbit	polyclonal	1:100	Sigma-Aldrich	G926
Iba-1	Rabbit	polyclonal	1:250	Wako Chemicals	019-19741
MMP-9	Goat	polyclonal	1:100	R&D Systems	AF909
NeuN	Rabbit	monoclonal	1:1000	Abcam	ab177487
Osteopontin (OPN/SPP1)	Goat	polyclonal	1:100	R&D Systems	AF808
PSD95	Rabbit	monoclonal	1:600	Abcam	ab76115
SCARA-1 (CD204)	Mouse	monoclonal	1:100	Bio-Rad (AbD Serotec)	MCA1322
Tubb3	Rabbit	monoclonal	1:500	BioLegend	845501
Tuj1	Mouse	monoclonal	1:1000	Abcam	ab14545
VGluT1	Guinea pig	polyclonal	1:6000	MilliporeSigma	AB5905
Secondary antibody					
Anti-goat IgG (Cy5)	Donkey	polyclonal	1:200	Jackson ImmunoResearch	705-175-147
Anti-guinea pig IgG (Cy3)	Donkey	polyclonal	1:200	Jackson ImmunoResearch	706-106-150
Anti-mouse IgG (Cy2)	Donkey	polyclonal	1:200	Jackson ImmunoResearch	715-225-151
Anti-mouse IgG (Cy3)	Donkey	polyclonal	1:200	Jackson ImmunoResearch	715-165-151
Anti-mouse IgG (Cy5)	Donkey	polyclonal	1:200	Jackson ImmunoResearch	715-175-151
Anti-rabbit IgG (Cy2)	Donkey	polyclonal	1:200	Jackson ImmunoResearch	711-225-152
Anti-rabbit IgG (Cy5)	Donkey	polyclonal	1:200	Jackson ImmunoResearch	711-175-152
Anti-rat IgG (Cy3)	Donkey	polyclonal	1:200	Jackson ImmunoResearch	712-166-153
Anti-rat IgG (DyLight 649)	Donkey	polyclonal	1:200	Jackson ImmunoResearch	711-405-152

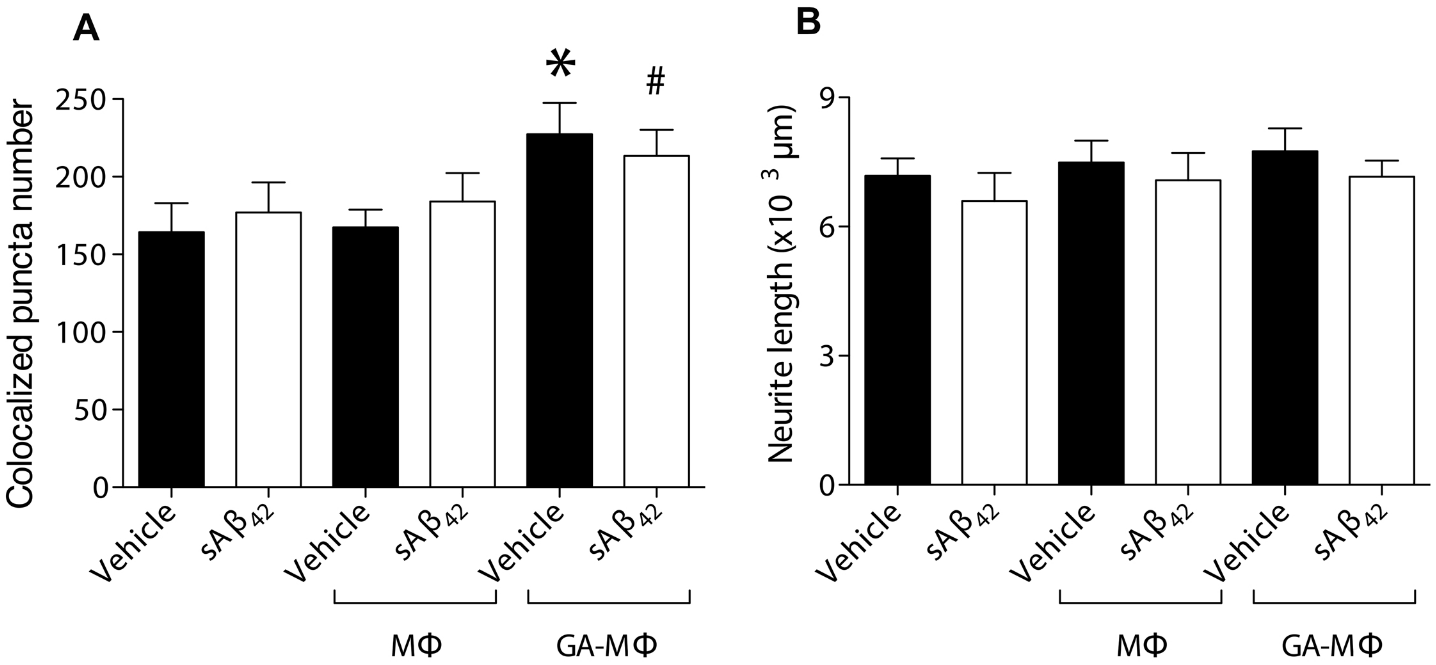

Supplementary Figure 1. Scrambled Aβ_{42} does not induce synaptic loss or neuritic retraction in primary cortical neurons. No statistical difference in synaptic puncta number (**A**) or neuritic length (**B**) was found between groups of vehicle and scrambled control Aβ (sAβ_{42}). Note significant increase in synaptic puncta number of the primary cortical neurons when co-culturing with GA-treated macrophages (GA-MΦ). Data expressed as mean ± s.e.m.; n = 48 fields analyzed from 3 independent experiments; * $P < 0.05$ or # $P < 0.05$ vs. vehicle or sAβ_{42} alone (no MΦ), respectively, by one-way ANOVA and Tukey's post-test.

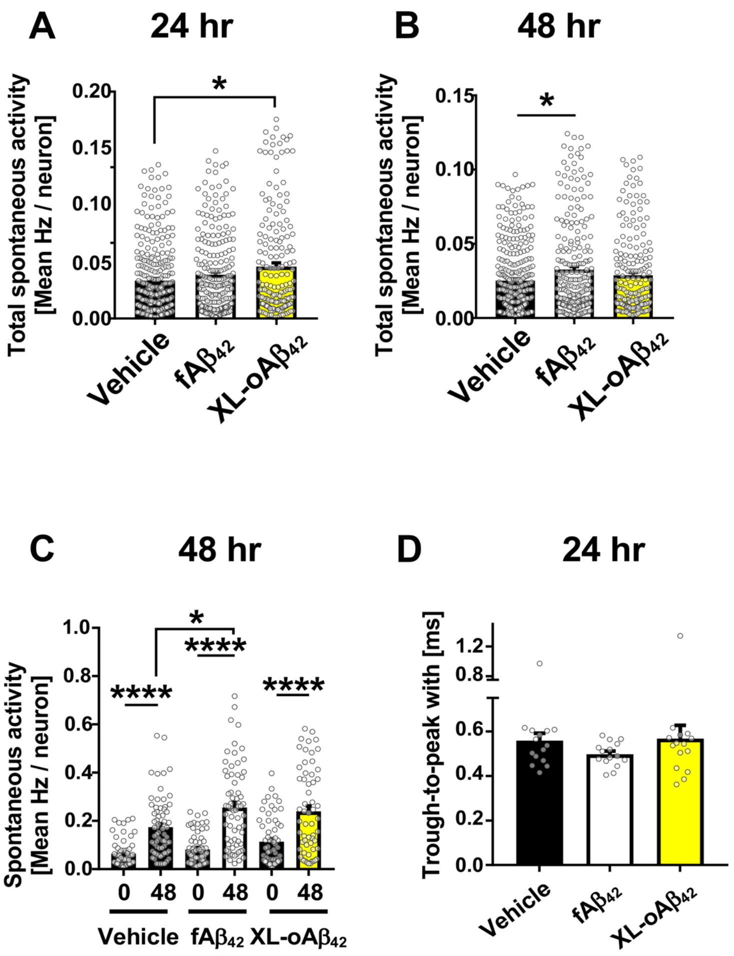

Supplementary Figure 2. Microelectrode array analysis of neuronal activity. (**A-B**) Frequency of total spontaneous activity recorded from individual neurons after 24 (**A**) and 48 (**B**) hours incubation with vehicle, $fA\beta_{42}$ or XL-$oA\beta_{42}$ (24 hrs: Kruskal-Wallis analysis of means by ranks $P = 0.0368$ followed by Dunn's multiple comparison test $P = 0.0248$; 48 hrs: Kruskal-Wallis analysis of means by ranks $P = 0.0429$ followed by Dunn's multiple comparison test $P = 0.0264$). (**C**) Comparison of spontaneous activity measured from the same individual neuron populations over time, from untreated (time 0) to 48 hours incubation with vehicle, $fA\beta_{42}$ or XL-$oA\beta_{42}$ (Kruskal-Wallis analysis of means by ranks $P = 0.0198$ followed by Dunn's multiple comparison test $P = 0.0192$ Vehicle to $fA\beta_{42}$). (**D**) Trough-to-peak width as measured after 24 hours incubation with vehicle, $fA\beta_{42}$ or XL-$oA\beta_{42}$ (not significant, One-Way ANOVA $P = 0.4108$). Data expressed as mean ± s.e.m. from 5 independent experiments; * $P < 0.05$; **** $P < 0.0001$.

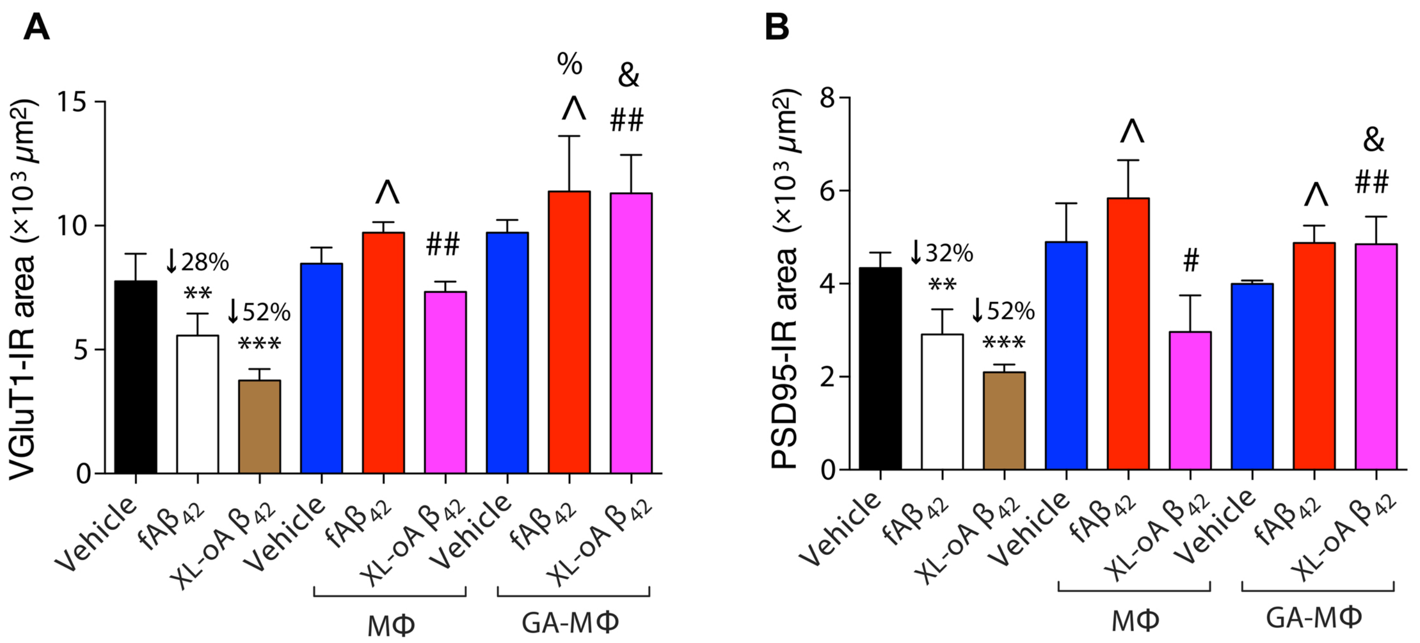

Supplementary Figure 3. MΦ and moreover GA-MΦ preserve pre(VGluT1)- and post(PSD95)-synaptic terminals in P1 neurons. Presynaptic VGluT1 (**A**) and postsynaptic PSD95 (**B**) areas were remarkably reduced with incubation of fAβ_{42} or oAβ_{42}. Note that the reduced synaptic density was significantly preserved by co-culturing with MΦ or GA-MΦ. Note significant increase in neuritic length of the primary cortical neurons when co-culturing with GA-MΦ. Data expressed as mean ± s.e.m.; n = 48 fields analyzed from 3 independent experiments; ** $P < 0.01$, *** $P < 0.001$ vs. vehicle; ^ $P < 0.01$ vs. fAβ_{42}; # $P < 0.05$, ## $P < 0.01$ vs. XL-oAβ_{42}; % $P < 0.05$ vs. MΦ + fAβ_{42}; & $P < 0.01$ vs. MΦ + XL-oAβ_{42}, by one-way ANOVA and Tukey's post-test.

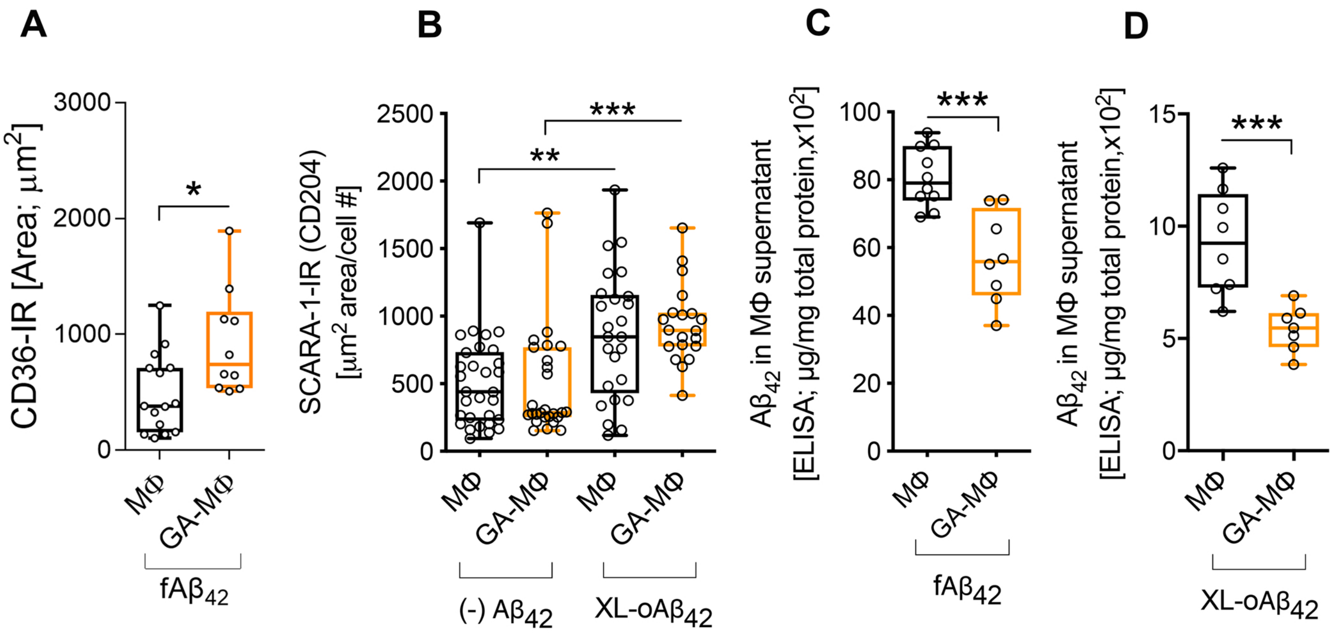

Supplementary Figure 4. Effects of GA on MΦ expression of CD36 and SCARA-1 in response to Aβ_{42} oligomers vs. fibrils and extracellular degradation. (**A**) GA increases expression of CD36 in MΦ following 30 min incubation with fAβ_{42}. (**B**) No difference is observed in SCARA-1 immunosignal between MΦ and GA-MΦ following XL-oAβ_{42} exposure. However, there is a significant increase in SCARA-1-IR expression in MΦ and GA-MΦ following XL-oAβ_{42}. (**C,D**) Decrease of Aβ_{42} levels in the supernatant after 30 min incubation. Note the significantly lower levels of Aβ_{42} in MΦ exposed to oAβ_{42} (**D**) compared to fAβ_{42} (**C**). Data expressed as mean ± s.e.m.; * $P < 0.05$, ** $P < 0.01$, *** $P < 0.001$ vs. untreated MΦ, by either Student's t-test for two-group comparison or one-way ANOVA and Tukey's post-test for four-group analysis.

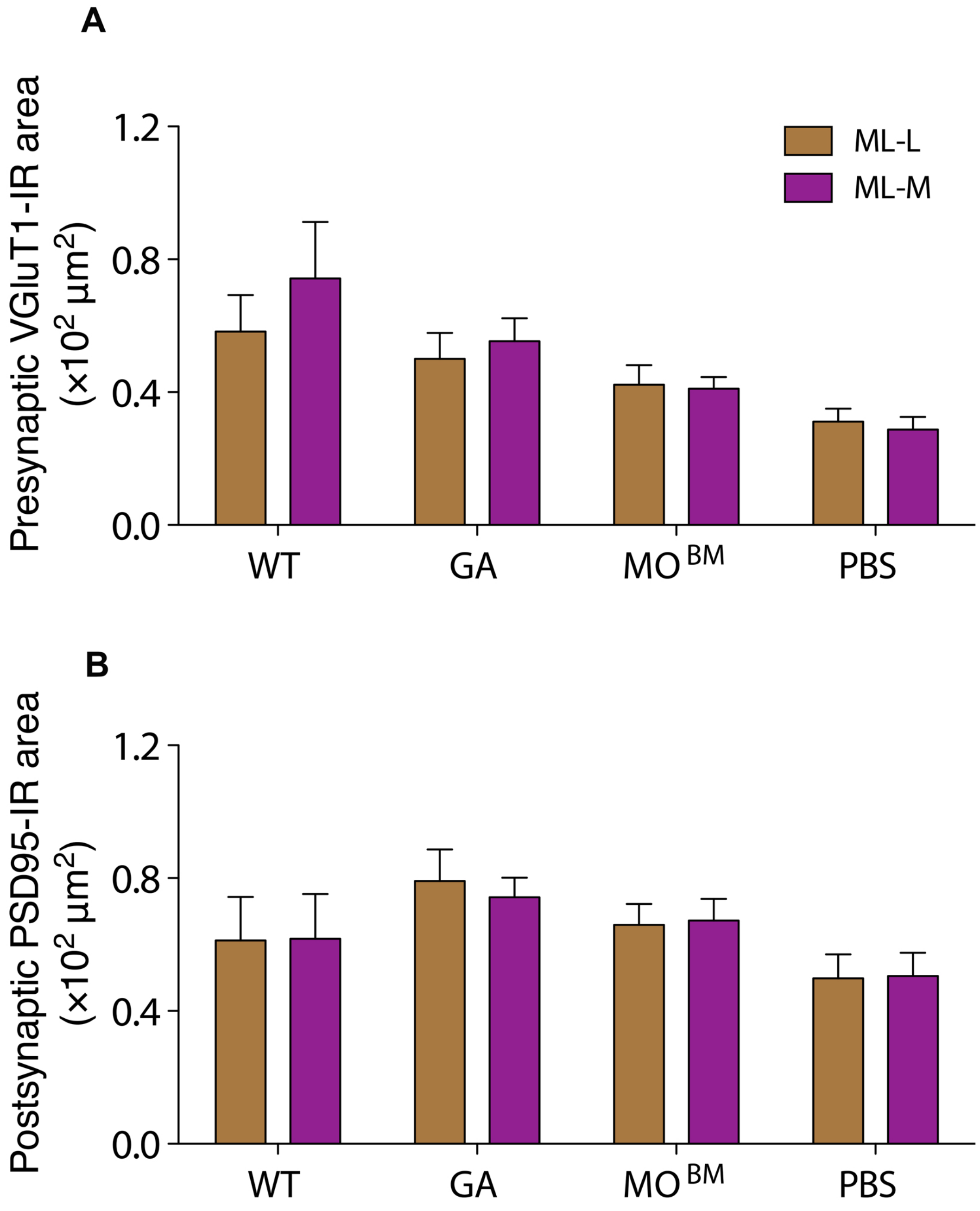

Supplementary Figure 5. Similar synaptic density in lateral blade molecular layer (ML-L) and medial blade molecular layer (ML-M) of the dentate gyrus across all experimental groups. (**A-B**) No significant difference in presynaptic VGluT1 immunoreactive (IR) area (**A**) or postsynaptic PSD95 area (**B**) was found between the lateral blade molecular layer (ML-L) and medial blade molecular layer (ML-M) of the dentate gyrus across all groups. Data expressed as mean ± s.e.m.; n = 6 mice per group; One-way ANOVA and Tukey's post-test was applied to compare between mean values in the ML-L vs. ML-M in the four experimental groups.

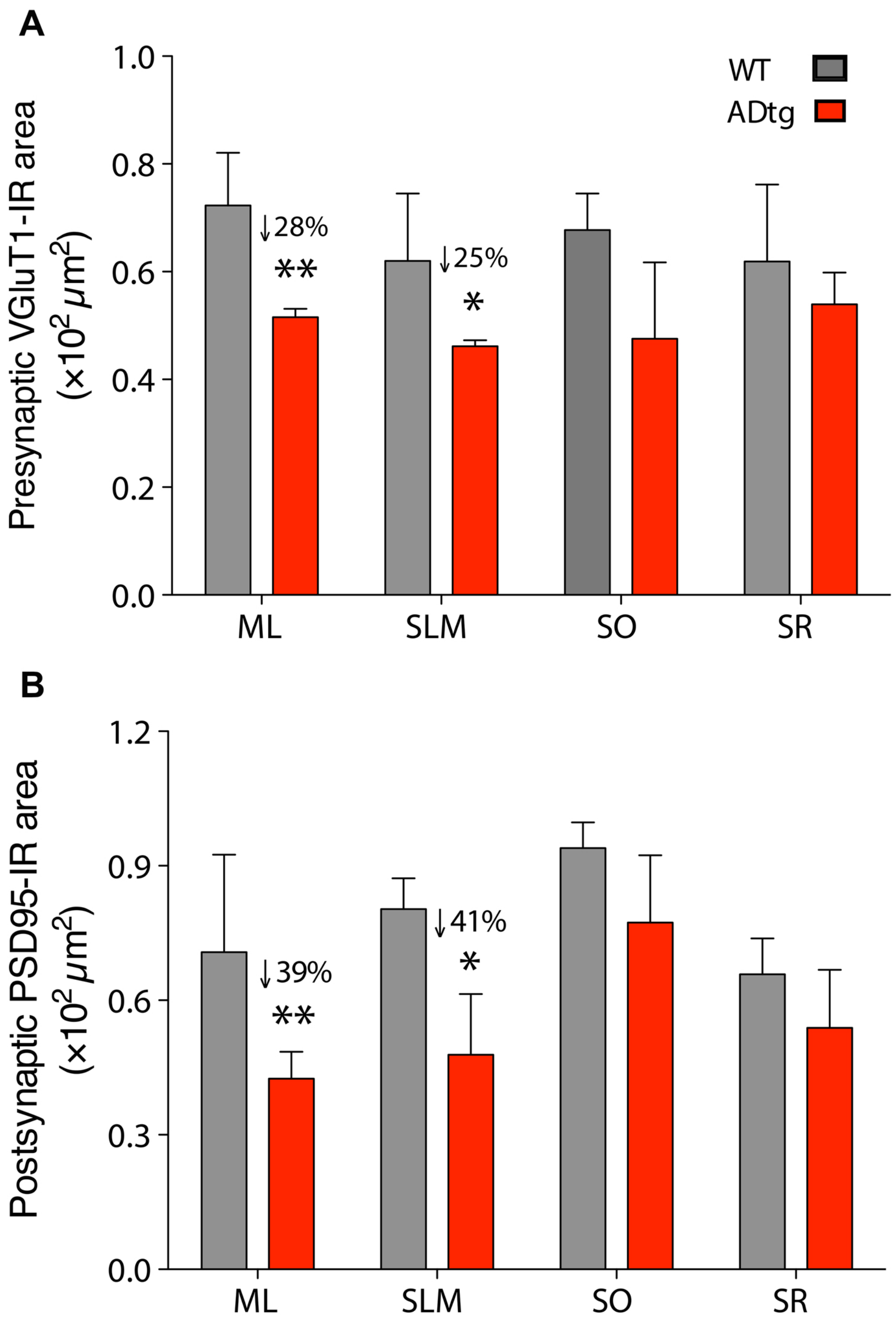

Supplementary Figure 6. Synaptic loss in hippocampal structures of 10-month-old male ADtg mice. Quantification of presynaptic area (**A**) and postsynaptic area (**B**) in the dentate gyrus and CA1 regions of WT and ADtg animals. Note the substantial synaptic loss in ML [molecular layers (ML) of dentate gyrus (DG)] and SLM (stratum lacunosum-moleculare), but not in stratum radiatum (SR) or stratum oriens (SO) of CA1 in the ADtg mice. Data expressed as mean ± s.e.m., with % reduction compared to WT mice; n = 6 mice per group; * $P < 0.05$, ** $P < 0.01$ compared to WT control mice, one-way ANOVA with Tukey's post-test.

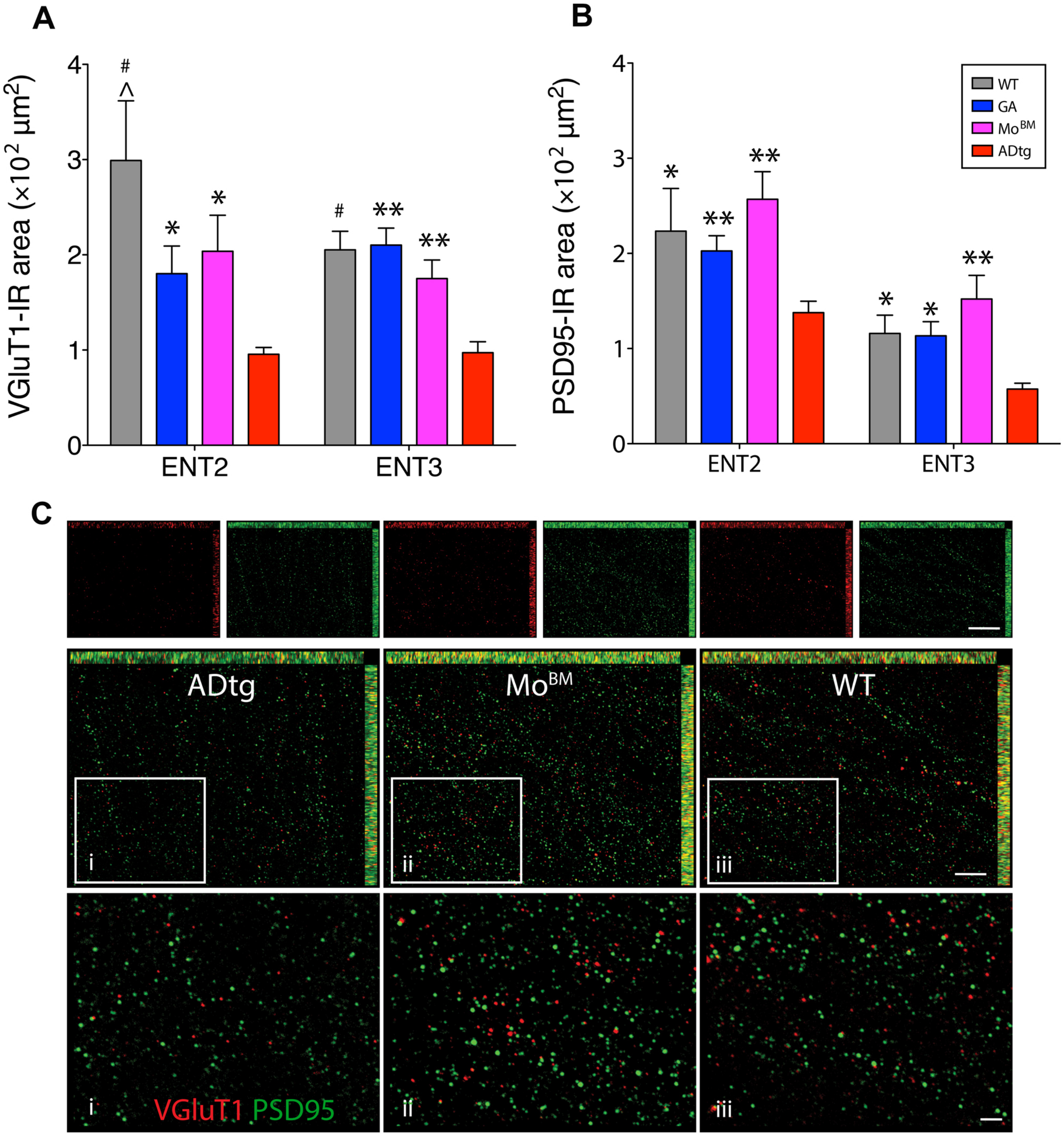

Supplementary Figure 7. Immunomodulation by GA or grafted monocytes preserves excitatory synapses in the brains of 13-months-old male ADtg mice. Quantification of presynaptic VGluT1 (**A**) and postsynaptic PSD95 (**B**) areas in ENT2/3 of ADtg and WT mice. Note the significant increase in presynaptic and postsynaptic areas in ENT2/3 following GA immunization or grafted Mo^{BM} in ADtg mice. Data expressed as mean ± s.e.m.; n = 6 mice per group; * $P < 0.05$, ** $P < 0.01$, *** $P < 0.001$, and # $P < 0.001$, compared to ADtg control; @ $P < 0.05$ compared to Mo^{BM}, one-way ANOVA with Tukey's post-test. (**C**) Representative high-resolution microscopic images of ENT2 from groups of ADtg, Mo^{BM} and WT mice, immunolabeled for VGluT1 and PSD95. The density of presynaptic or postsynaptic areas was greater in WT (**iii**) and Mo^{BM}-immunized (**ii**) mice than in PBS-injected control ADtg mice (**i**). Scale bars represent 20 µm (upper), 10 µm (middle) and 2 µm (lower), respectively.

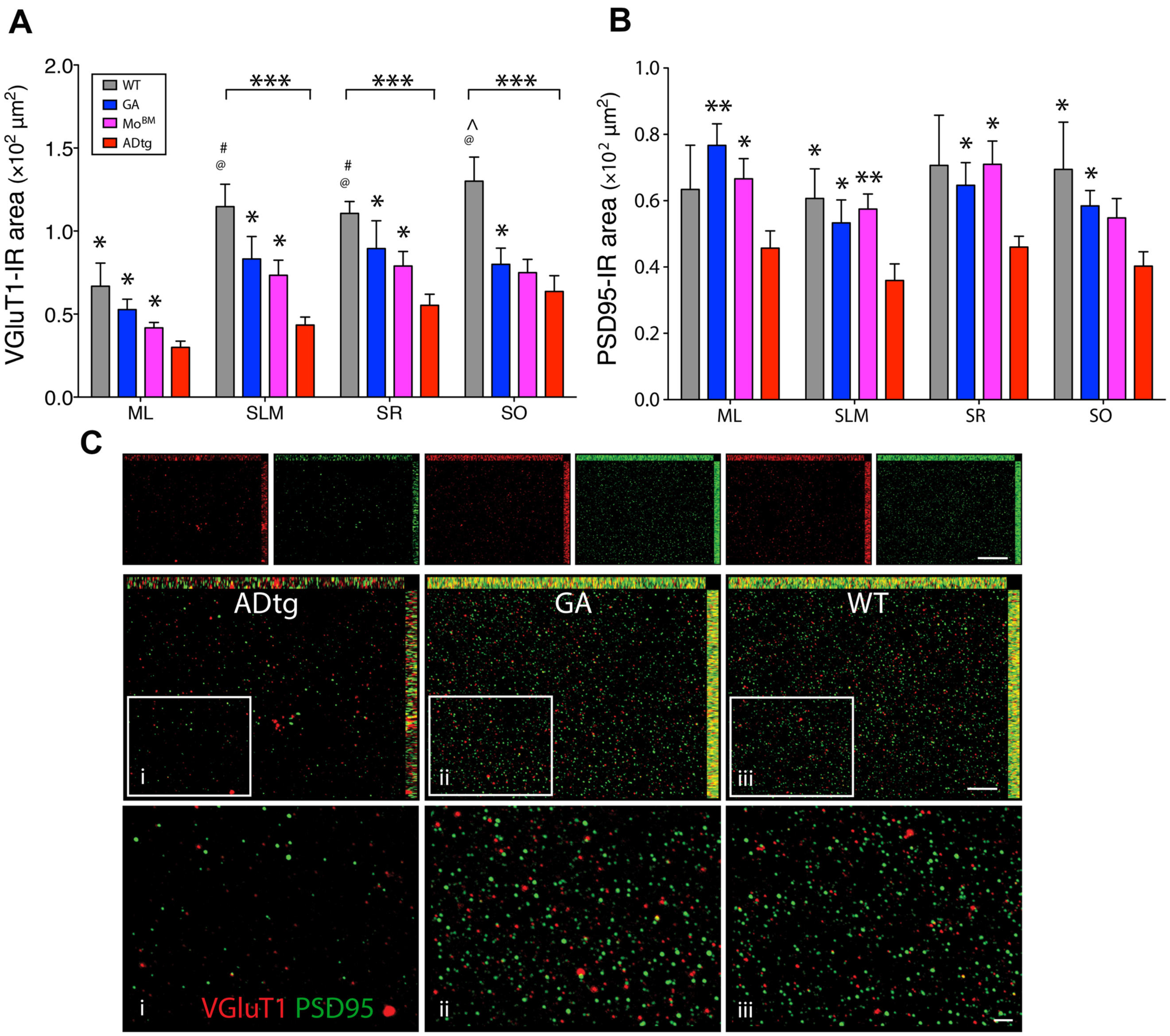

Supplementary Figure 8. Preserved synapses in substructures of HIPPO by GA immunization and grafted monocytes is associated with attenuated disease progression in 13-months-old male ADtg mice. (**A**) Quantification of presynaptic area in DG and CA1 from ADtg and WT mice. Note significantly greater presynaptic VGluT1-IR area in ML, SLM and SR in the groups of GA and Mo^{BM} treated mice compared to ADtg controls. (**B**) Quantification of postsynaptic area in DG and CA1 across all experimental mouse groups. Greater postsynaptic PSD95-IR area in ML, SLM, SR and SO noted in GA-immunized mice compared to ADtg controls. Data expressed as mean ± s.e.m. n = 6 mice per group; * $P < 0.05$, ** $P < 0.01$ and *** $P < 0.001$, compared to ADtg; @ $P < 0.05$ compared to Mo^{BM}. And ^ $P < 0.05$ compared to ADtg mice. (**C**) Representative high-resolution microscopic images of ENT2 from groups of ADtg, GA and WT mice, immunolabeled for VGluT1 and PSD95. The density of presynaptic or postsynaptic areas was greater in WT (**iii**) and GA-immunized (**ii**) mice than in PBS-injected control ADtg mice (**i**). Scale bars represent 20 µm (upper), 10 µm (middle) and 2 µm (lower), respectively.

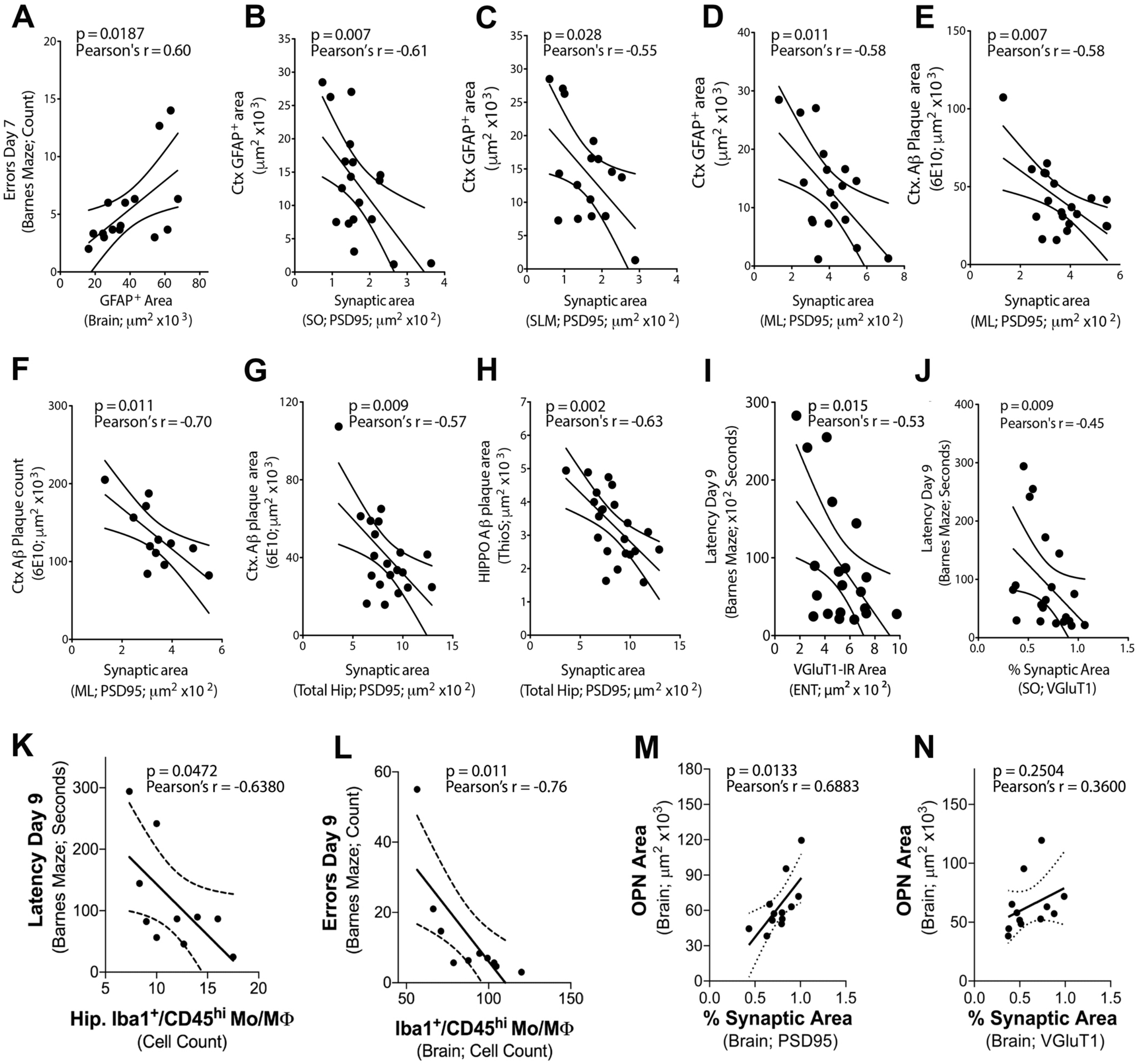

Supplementary Figure 9. Correlations between synaptic integrity, neuropathology, cognitive function and macrophage infiltration in 13-months-old male ADtg mice. (**A**) Direct correlation between astrogliosis ($GFAP^+$) and number of errors on day 7 of the Barnes maze test. (**B-D**) Inverse correlations between postsynaptic PSD95 area in SO, SLM, ML and cerebral $GFAP^+$ areas. (**E-H**) In the hippocampal substructures, postsynaptic areas negatively correlate with Aβ plaque burden. (**I-J**) Synaptic integrity, measured by presynaptic VGluT1 area in ENT and percentage of presynaptic area in SO, predict cognitive functions measured by latency on day 9 of Barnes maze. (**K-L**) Hippocampus and brain $Iba\text{-}1^+/CD45^{hi}$ Mo/MΦ cell counts correlate with improved cognitive status measured by latency and error count on day 9 of Barnes maze. (**M-N**) Direct and linear association between cerebral MΦ-derived OPN/SPP1 expression and postsynaptic (**M**) but not presynaptic (**N**) integrity. Correlation analyses were carried out using the Pearson's coefficient *r* test. Group mean ± s.e.m. are shown; n = 10-22 mice per analysis.
